# Improvement of the LbCas12a-crRNA System for Efficient Gene Targeting in Tomato

**DOI:** 10.3389/fpls.2021.722552

**Published:** 2021-08-10

**Authors:** Tien Van Vu, Duong Thi Hai Doan, Mil Thi Tran, Yeon Woo Sung, Young Jong Song, Jae-Yean Kim

**Affiliations:** ^1^Division of Applied Life Science (BK21 FOUR Program), Plant Molecular Biology and Biotechnology Research Center, Gyeongsang National University, Jinju, South Korea; ^2^National Key Laboratory for Plant Cell Biotechnology, Agricultural Genetics Institute, Hanoi, Vietnam; ^3^Crop Science and Rural Development Division, College of Agriculture, Bac Lieu University, Bạc Liêu, Vietnam; ^4^Division of Life Science, Gyeongsang National University, Jinju, South Korea

**Keywords:** LbCas12a, gene targeting, gene editing, homology-directed repair, SpCas9, geminiviral replicon

## Abstract

Plant gene targeting (GT) can be utilized to precisely replace up to several kilobases of a plant genome. Recent studies using the powerful clustered regularly interspaced short palindromic repeats (CRISPR) and CRISPR-associated (Cas) nucleases significantly improved plant GT efficiency. However, GT for loci without associated selection markers is still inefficient. We previously utilized *Lachnospiraceae bacterium* Cas12a (LbCas12a) in combination with a replicon for tomato GT and obtained high GT efficiency with some selection markers. In this study, we advance our GT system by inhibiting the cNHEJ pathway with small chemical molecules such as NU7441. Further optimization of the GT is also possible with the treatment of silver nitrate possibly via its pronounced actions in ethylene inhibition and polyamine production. Importantly, the GT efficiency is significantly enhanced with the use of a temperature-tolerant LbCas12a (ttLbCas12a) that is capable of performing target cleavage even at low temperatures. Targeted deep sequencing, as well as conventional methods, are used for the assessment of the editing efficiency at both cell and plant levels. Our work demonstrates the significance of the selection of gene scissors, the appropriate design and number of LbCas12a crRNAs, the use of chemical treatments, and the establishment of favorable experimental conditions for further enhancement of plant HDR to enable efficient GT in tomato.

## Introduction

Plant gene targeting (GT) was first reported in Paszkowski et al. (1988), although that study only obtained low efficiency of targeting, and GT usually requires at least one associated selection marker for the practical achievement of GT events (Puchta and Hohn, 2005; Van Vu et al., 2019). Without any targeted double-stranded break (DSB) and with an antibiotic selection marker, the GT efficiency remained extremely low (Terada et al., 2002). An important improvement was made to plant GT by introducing DSBs at the targeted sites using a pre-inserted I-SceI recognition sequence; this approach ultimately enhanced the GT efficiency up to hundreds of fold (Puchta et al., 1993, 1996), but the efficiency was still low (i.e., up to 1.83%) even it required two selection markers. The subsequent development of the first, second and, especially, third generations of site-directed nucleases (SDNs) has revolutionized precision gene-editing technology with the easy customization of targeted DSBs at any site of interest for exchanging donor DNA templates (Wright et al., 2005; Shan et al., 2013; Zhang et al., 2013).

Until recently, SDN-based GT efficiency has been significantly enhanced through combinations of SDN complexes and geminiviral replicons, which are autonomously replicative vectors for supplying high doses of homologous DNA template to DSB repair foci (Cermak et al., 2015; Butler et al., 2016; Gil-Humanes et al., 2017; Vu et al., 2020). Further improvement of plant GT was also possible with the suppression of canonical non-homologous end joining (cNHEJ) (Qi et al., 2013; Maruyama et al., 2015; Robert et al., 2015; Endo et al., 2016) or activation of homology-directed repair (HDR) mechanisms (Song et al., 2016) using biological approaches or chemical treatments. Nevertheless, chemical treatments have not been heavily studied in conjunction with clustered regularly interspaced short palindromic repeats (CRISPR)-Cas-based GT in plants. Overall, the practical GT efficiency with allele-associated selection markers was approximately 10% in most of the “accessible” plants and considerably lower in difficult systems or those with targeted loci lacking selection markers (Van Vu et al., 2019). Therefore, continuous improvement of plant GT remains necessary, especially for applications in less accessible plants.

Our previous work showed the significant improvement of plant GT using geminiviral replicons and LbCas12a, rather than SpCas9 (Vu et al., 2020). More importantly, the activity of LbCas12a nucleases was more temperature-dependent than that of SpCas9 (Malzahn et al., 2019; Vu et al., 2020). Recently, a temperature-tolerant LbCas12a mutant (D156R), known as ttLbCas12a, which exhibited significantly improved cleavage activity and hence GT efficiency, was reported in *Arabidopsis* using floral dipping-based gamete stage transformation (Merker et al., 2020a; Schindele and Puchta, 2020). However, further characterization of ttLbCas12a for somatic cell-based GT systems such as tomato GT, especially in combination with DNA replicons, has not been undertaken. We hypothesized that ttLbCas12a could be utilized to further improve our replicon-based plant GT system through appropriate customization of its nuclease-crRNA complexes. Chemical treatment for suppressing cNHEJ or activating HDR pathways was also tested and validated for practical GT applications in tomato. In this report, we describe a further comparison of LbCas12a and SpCas9 in inducing GT at the same specific SlANT1 locus. Subsequently, extensive characterization of ttLbCas12a in comparison with LbCas12a is shown at the SlANT1, SlHKT1;2, and SlEPSPS1 loci with or without allele-associated selection markers. Our work demonstrates the significance of utilizing the appropriate design of CRISPR-Cas/crRNA complexes, chemical treatments, and favorable experimental conditions to further enhance plant HDR for efficient GT in tomato.

## Results

### NU7441 Treatment Enhances LbCas12a-Based GT Efficiency

A plant genomic DNA DSB may be repaired by two major competing mechanisms, namely, cNHEJ and HDR. The GT approach is based on the HDR mechanism; therefore, to increase its efficacy, blocking the cNHEJ pathway is a good option (Van Vu et al., 2019). A number of studies have been published regarding the uses of chemical treatments for blocking cNHEJ to enhance GT efficiency in mammals (Chu et al., 2015; Maruyama et al., 2015; Robert et al., 2015; Zhang et al., 2017). However, only limited information regarding the applications of the chemicals to plant GT is currently available. Therefore, we selected SCR7 (an inhibitor of DNA ligase IV), NU7441 [a DNA-dependent protein kinase (DNA-PKcs) inhibitor], and KU0060648 (a dual inhibitor of DNA-PKcs and phosphatidylinositol-3 kinase (PI-3K)) to determine their effects on plant GT. Previously, a replicon-based CRISPR-Cas-mediated targeted DNA insertion system was successfully developed with SlANT1 as a visible marker (Cermak et al., 2015; Vu et al., 2020). We used SpCas9 (pTC217) and LbCas12a (pHR01 and pMR01) carrying GT vectors to assess the effects of chemical treatments around the replicon peak (3–5 days post-transformation) and GT scoring followed Vu et al. (2020) since the scoring total GT callus number provides richer data than simply scoring GT cotyledon number.

**FIGURE 1:**
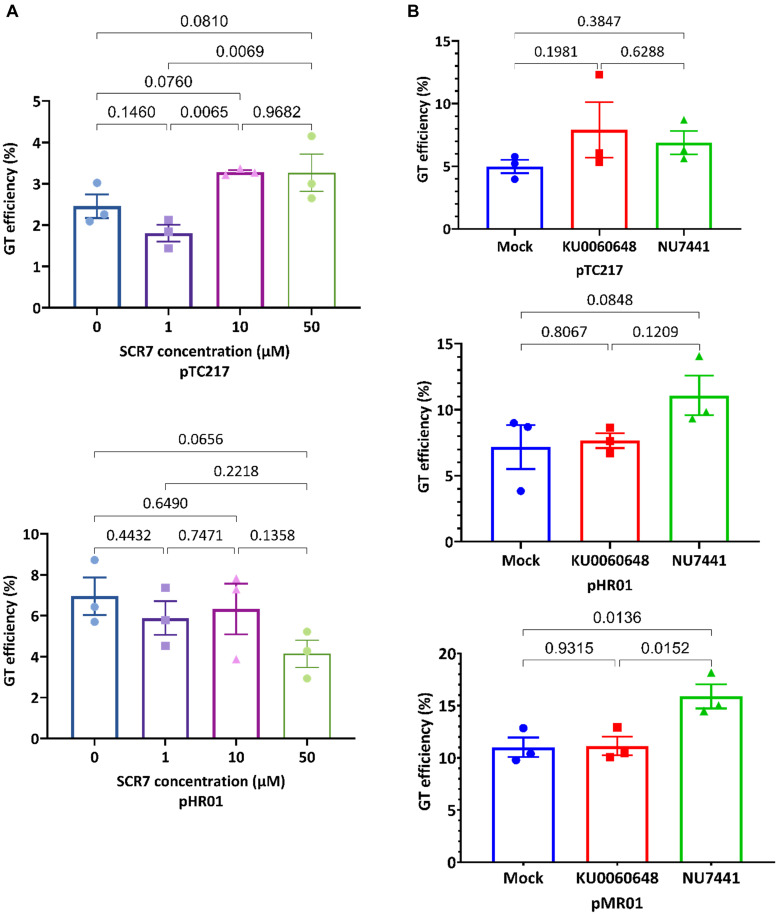
Enhancement of GT efficiency by chemical treatments for blocking cNHEJ. **(A)** SpCas9 (pTC217, top panel) and LbCas12a (pHR01, bottom panel)-based GT efficiency obtained from the treatment of different SCR7 concentrations. SCR7 was added to the non-selection medium (NSEL), and the explants were incubated for 5 days before transferring to the selection medium (SEL5). The GT efficiencies were calculated at 21 dpt. **(B)** The impacts of KU0060648 and NU7441 on CRISPR/Cas-based GT. SpCas9-based pTC217 (top panel) and LbCas12a-based pHR01 (middle panel) were cloned with single geminiviral replicons, whereas LbCas12a-based pMR01 (bottom panel) was released from a multireplicon vector. The chemical was added to the non-selection medium (NSEL), and the explants were incubated for 5 days before transferring to the selection medium (SEL5). The GT efficiencies were calculated at 21 dpt. Multiple comparisons of the means and plotting were conducted by GraphPad Prism version 9 using one-way ANOVA and Fisher’s LSD test. The *p*-values of each compared mean pair are shown on the top of the bars.

During the GT reaction performed using the SpCas9-based pTC217 vector, treatment with the DNA ligase IV inhibitor led to an improvement in the GT efficiency compared to the mock control (Figure 1A, upper panel). The GT efficiency was increased to around 3.3% with the treatments of either 10 or 50 μM SCR7 compared to the mock 0 μM treatment or the 1 μM SCR7 treatment (Supplementary Table 1). However, Fisher’s LSD test for comparison of the GT efficiency between the treated concentrations and the mock control did not return significant *p*-values to determine whether the GT enhancement was strong enough (Figure 1A, upper panel). The data indicate that 10 μM SCR7 was the best concentration for SpCas9-based GT in tomato. By contrast, the LbCas12a-based pHR01 construct exhibits a decreasing trend from the mock control to the highest SCR7 concentration. There were mild GT efficiency changes among the mock and 1 or 10 μM SCR7 treatments. Nevertheless, the GT efficiency was dramatically reduced to 4.14 ± 0.66% at 50 μM SCR7 from 6.95 ± 0.91% in the mock treatment (68% reduction) (Figure 1A, bottom panel and Supplementary Table 1). Our observation during the experiment revealed a mildly negative impact of the SCR7 on the viability of the explants at 1 and 10 μM, but more severe at the 50 μM concentration.

To test the impacts of NU7441 and KU0060648 on CRISPR-Cas-based GT in tomato, we employed both single and multiple replicon systems to carry the SpCas9 or LbCas12a constructs. In the case of SpCas9, the GT efficiency was slightly increased (Figure 1B, top panel) when 0.2 μM KU0060648 or 1 μM NU7441, which were within the optimal concentration ranges tested in human cells (Robert et al., 2015), was applied to NSEL medium (Supplementary Figure 1) and incubated from days 3 to 8 post-transformation. However, pairwise comparison resulted in *p*-values that were not small enough to reject the null hypothesis. A similar situation also occurred for the LbCas12a-based single replicon (pHR01), although NU7441 treatment led to a smaller *p*-value. In this case, 1 μM NU7441 treatment resulted in GT efficiency at 11.07 ± 1.50% compared to 7.17 ± 1.67% of that of the mock control, which represented a 1.54-fold enhancement (Figure 1B, middle panel and Supplementary Table 2). The effect is considerably more clear with NU7441 treatment using the LbCas12a-based multiple replicon system (pMR01). Blocking cNHEJ with 1 μM NU7441 significantly increased the multireplicon-based GT efficiency from 11.01 ± 0.93% in the mock control to 15.89 ± 1.15%, representing an approximately 1.44-fold change (Figure 1B, bottom panel and Supplementary Table 2). There was almost no GT efficiency change in the case of KU0060648 treatment using both the single- and multi-replicon.

### Silver Nitrate Treatment Enhances GT Efficiency and Purple Shoot Regeneration

Recently, polyamines, such as putrescine, spermidine, and spermine, were shown to enhance HDR by facilitating RAD51-mediated homologous strand annealing and synaptic complex formation in mice (Lee et al., 2019). We added 1 mM putrescine, spermidine, or spermine to the NSEL medium and incubated the transformed explants for 5 days after cocultivation with agrobacteria carrying the multireplicon pMR01 plasmid. In contrast to the observations made in animals, no improvement in GT efficiency was achieved by any of the polyamines in the two replicates (Supplementary Table 3). In fact, the GT efficiency was reduced with supplementation with longer chain polyamines (i.e., spermidine and spermine) (Supplementary Figure 2). We also observed a higher proliferation of the purple GT calli and a delay in shoot formation from the explants treated with spermidine or spermine compared to the mock control. We surmised that direct supplementation with synthetic polyamines, especially spermidine and spermine, at high concentrations might not facilitate organ regeneration. Thus, we next used silver nitrate (AgNO_3_) to increase endogenous polyamines. Silver nitrate was shown to stimulate the activity of arginine decarboxylase (ADC; EC 4.1.1.9), one of the key enzymes of putrescine and polyamine biosynthesis (Hanfrey et al., 2001). Using the multireplicon pMR01 vector for this experiment, we treated the transformed explants with 30 μM AgNO_3_ for 5 days on NSEL medium. In two replicates, we observed a significant increase in the number of purple GT spots per explant compared to that of the mock control at 21-day post-transformation (dpt) (Supplementary Figure 3A and Supplementary Table 4). Furthermore, the addition of AgNO_3_ at a later stage, in the first subculture with the SEL4C medium, stimulated shoot regeneration from purple calli (Supplementary Figure 3B). This finding is consistent with that silver nitrate enhances somatic embryogenesis (Roustan et al., 1990; Giridhar et al., 2004).

### LbCas12a-Based GT Is Superior to the SpCas9-Based GT

**FIGURE 2:**
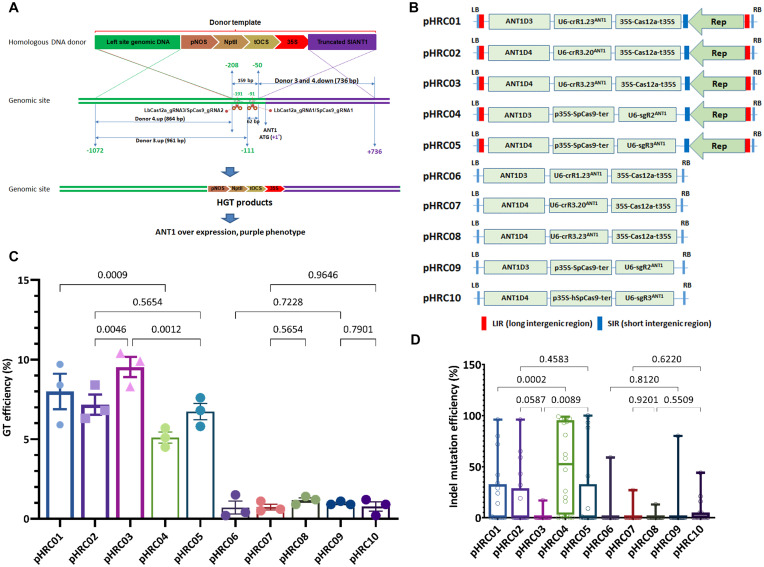
Performance of the LbCas12a and SpCas9 nucleases in GT-mediated DNA insertion in tomato. **(A)** Schematic diagram of CRISPR/Cas-based GT processes. The SlANT1 genomic site was cleaved by the CRISPR/Cas complexes at the positions of LbCas12a_gRNA1 and 3 and SpCas9_gRNA1 and 2, denoted by scissors. Subsequent repairs of the DSBs were conducted with the addition of donor templates that contain upstream homologous arms (corresponding to Donor 3.up and Donor 4.up) and downstream truncated SlANT1 (corresponding to Donors 3 and 4.down) of the DSB sites and the inserted sequences containing the kanamycin selection marker (pNOS-NptII-tOCS) followed by a CaMV 35S promoter (35S) for constitutively driving SlANT1 expression. The lengths in bp of the homologous arms are also shown. The distances in bp among the cleaved sites and the starts and ends of the donor sequences were calculated and illustrated in relation to the ATG start codon of the SlANT1 gene with the A as the +1 position. The sequence upstream of the SlANT1 start codon is drawn by the green lines, and purple lines are drawn for the downstream part. The crossing discontinuous lines between the homologous DNA donor and genomic site depict the expected homologous recombination for sequence exchanges. Successful GT would integrate the selection marker and 35S promoter at the DSB sites, thereby supporting event selection and screening by kanamycin antibiotic and purple phenotype. **(B)** Binary vectors used for comparison of SpCas9- and LbCas12a-based GT performance. Each vector contained a homologous donor described in panel **(A)** and the Supplementary Data 1, an expression cassette of sgRNA/crRNA and SpCas9 or LbCas12a expression cassette. Two sets of vectors were used: only T-DNA and replicon-based systems for comparison. **(C)** Scatter dot-bar plots showing the GT efficiencies of the tested constructs. **(D)** Boxplot showing the indel mutation efficiencies of the GT constructs at the plant stage. The GT efficiencies were calculated at 21 dpt. Multiple comparisons of the means and plotting were conducted by GraphPad Prism version 9 using one-way ANOVA and Fisher’s LSD test. The *p*-values of each compared mean pair are shown on the top of the bars.

In our previous report, LbCas12a was shown to mediate GT more effectively than the SpCas9 system. The comparisons were conducted at the SlANT1 locus under various experimental conditions (Vu et al., 2020). However, the comparison using the single replicon-based pTC217 and pHR01 constructs might have flawed parameters, such as slightly different SSN-mediated binding and cutting sites and donors. Furthermore, the promoter and terminator driving the expression of SpCas9 and LbCas12a might also contribute to the differences. To better characterize and compare the GT performance of SpCas9 and LbCas12a, we designed a GT system with specific SlANT1 cutting sites that are accessible to both nucleases (Figure 2A and Supplementary Figure 4) and the same promoter and terminator to drive the transcription of Cas nucleases. The comparisons were conducted using single sgRNAs (sgR2^ANT1^ vs. crR1.23^ANT1^ and crR3.20^ANT1^, sgR3^ANT1^ vs. crR3.23^ANT1^) (Figure 2B and Supplementary Data 1). The 20-nt cRNA was also used for the LbCas12a (crR3.20^ANT1^) to mimic the exact binding sequences of the SpCas9 gRNAs (Supplementary Figure 4). Each of the GT tools was expressed from both the replicon and T-DNA to compare the GT efficiency of the two delivery methods (Figure 2B). Our data demonstrate the superiority of the replicon system compared to the T-DNA in plant GT, as the GT efficiencies were enhanced fivefold to eightfold with the replicons compared to that of the T-DNA tools (Figure 2C and Supplementary Figure 5).

Moreover, in keeping with our previous data (Vu et al., 2020), all of the LbCas12a-based GT constructs, except the 20-nt crRNA, exhibited significantly higher GT efficiencies than the SpCas9-based GT tools (Figure 2C, 8.00 ± 1.11% of pHRC01 vs. 5.10 ± 0.35% of pHRC04, 9.53 ± 0.63% of pHRC03 vs. 6.73 ± 0.52% of pHRC05). These results also indicate that the 23-nt crRNA LbCas12a mediated GT more effectively than the 20-nt crRNA (pHRC03 vs. pHRC02). Surprisingly, analysis of the indel mutation efficiency of the guide RNAs at the plant stage demonstrated a reverse correlation between the indel mutation efficiency and the GT efficiency at cutting site 1 and cutting site 2 (Figure 2D, pHRC01 vs. pHRC04, and pHRC03 vs. pHRC05 and Supplementary Figure 5). This result indicates that a considerably stronger indel mutation activity may negatively affect the GT reactions. Since cNHEJ-mediated indel mutations are favored throughout the life cycle of the cells and HDR is limited to the S-G2 phases (Van Vu et al., 2019), DNA DSBs that form at cell cycles other than S-G2 may lead to permanent modifications at the cutting sites, resulting in the inhibition of any further cleavage at these sites in the HDR-favorable cell cycles. Additionally, or alternatively, although relatively low indel-forming activity of LbCas12a compared to SpCas9, recurrent cutting by LbCas12a exposes more frequently the 3′ends of broken DNA, which initiate to copy donor template, consequently enhancing GT frequency. Our data confirmed the stronger activity of SpCas9 in generating DNA DSBs compared to that of LbCas12a in tomato (Figure 2D and Supplementary Figure 5). In the case of the pHRC02 and pHRC03 constructs, the indel efficiency of 20-nt crRNA seemed to higher compared to that of the 23-nt crRNA at the same cutting site but not at the significant *p*-value (0.0587) (Figure 2D). The highest indel mutation rate was 49.5% on average with sgR2^ANT1^ (Supplementary Figure 5). Most of the mutation traces appear to be deletions (Supplementary Figure 6). In the experiment, we obtained GT events with a typical purple phenotype due to the overexpression of SlANT1 and the subsequent accumulation of anthocyanin in the plants (Supplementary Figure 7).

### ttLbCas12a-Based GT Efficiency Was Better Than That of the Wild-Type Variant in the Case of the Dual crRNA System

**FIGURE 3:**
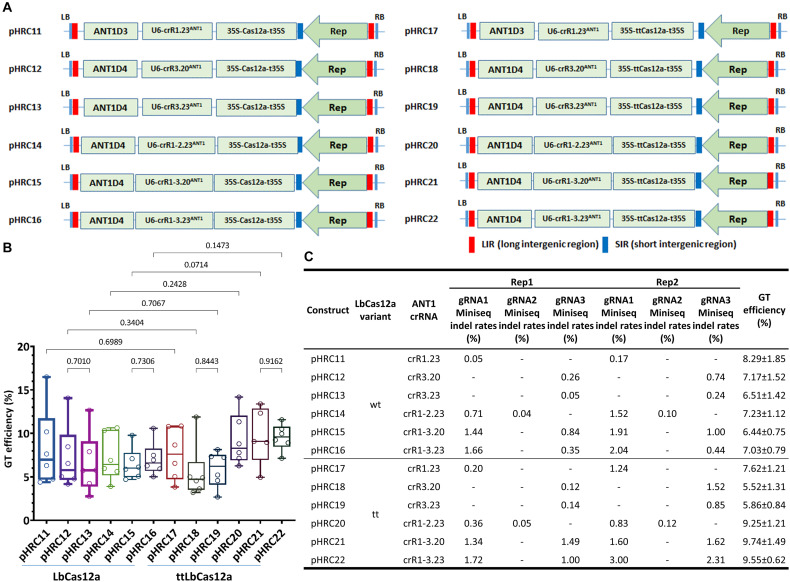
Comparison of GT efficiency between LbCas12a and ttLbCas12a at the SlANT1 locus. **(A)** Binary constructs with the same crRNAs and donors for the assessment of the GT efficiency of LbCas12a (left panel) and ttLbCas12a (right panel). **(B)** Boxplot showing the distributions of GT efficiency among the tools using various crRNAs with LbCas12a and ttLbCas12a. Multiple comparisons of the means of GT efficiency of the constructs using the same sets of crRNAs and donors but with LbCas12a or ttLbCas12a were conducted using Fisher’s LSD test, and the *p*-values are shown above the compared boxes. **(C)** Indel mutation rates induced by the Cas-crRNA complexes that were assessed at 10 dpt by targeted deep sequencing method. The GT efficiencies are also added for comparison.

The cleavage activity of Cas nucleases was shown to be temperature-dependent, especially in the case of LbCas12a (Malzahn et al., 2019). Previously, we showed the temperature dependency of CRISPR-Cas-mediated GT in tomato (Vu et al., 2020). In that case, LbCas12a exhibited considerably better GT support at temperatures as high as 31°C compared to that at 19°C or 25°C. This result partially explained why SpCas9 was superior in indel mutation formation compared to LbCas12a at RT. Recently, Puchta’s group reported a temperature-tolerant LbCas12a variant that significantly enhanced indel mutation (Schindele and Puchta, 2020) and GT (Merker et al., 2020a; Huang et al., 2021) efficacy in plants. The group used 24-nt single crRNAs that were released by ribozyme flanking sequences. It is interesting to characterize and compare the ttLbCas12a nuclease using our replicon system and on somatic cells of tomato. Here, we used a multiplexed editing approach with a single array of crRNAs and the gRNA length may affect the processing activity of the LbCas12a (Zetsche et al., 2017). We investigated the impacts of single and dual cleavages using 20- or 23-nt crRNA on the GT performance of both the wild-type and ttLbCas12a variants at the well-characterized SlANT1 locus (Figure 3A, Supplementary Figure 4, and Supplementary Data 1). To contribute to the comparison data, another dual crRNA (crR1-2.23^ANT1^) was also tested with LbCas12a_gRNA2 (Supplementary Figure 4), which was previously used by Vu and coworkers (Vu et al., 2020). Our data collected from 6 biological replicates were processed and compared using uncorrected Fisher’s LSD test. The statistical analysis demonstrated very mild changes in GT efficiency among the GT constructs using single crRNAs with both LbCas12a variants (Figure 3B; pHRC11, pHRC12, and pHRC13 for LbCas12a; and pHRC17, pHRC18, and pHRC19 for ttLbCas12a). The ttLbCas12a-based dual crRNA constructs (pHRC20, pHRC21, and pHRC22) showed higher GT efficiencies than LbCas12a (pHRC14, pHRC15, and pHRC16, respectively), although the *p*-values were only close to 0.05 and significant (Figure 3B). The highest difference in GT efficiency was found between ttLbCas12a-containing pHRC21 (9.74 ± 1.49%) and LbCas12a-containing pHRC15 (6.44 ± 0.75%) with crR1-3.20^ANT1^, exhibiting a 1.51-fold change (*p* = 0.0714) (Figures 3B,C).

In parallel with this experiment, we utilized targeted deep sequencing to analyze the cleavage activity of the nucleases with each of the gRNAs (Figure 3C) at 10 dpt. Due to the large size (∼0.3 × 0.3 cm) of the cotyledon explants used in the previous study, the majority of the cells were not in direct contact with the agrobacteria and thus were not all transformed. Therefore, we reduced the size of the cotyledon explant to ∼0.1 × 0.3 cm (Supplementary Figure 8) to reduce the untransformed cell portion for assessing the editing efficiency by targeted deep sequencing. The data collected from two biological replicates showed strong enhancement of indel mutation efficiencies of a gRNA if it was used in a dual crRNA construct, regardless of the LbCas12a variants. The largest enhancement was from LbCas12a_gRNA1 (23 nt) in the single crRNA crR1.23^ANT1^-carrying pHR11 (0.05%) and the dual crRNA crR1-3.23^ANT1^-expressing pHRC16 (1.66%) in replicate 1, representing a 33.2-fold increase (Figure 3C). The cleavage activity of ttLbCas12a was also higher than that of the WT variant when single cuts were used. For example, in the case of the crR1.23^ANT1^-expressing constructs, the indel mutation efficiency was 5.00- to 7.29-fold increased with ttLbCas12a (pHRC17) compared to LbCas12a (pHRC11) (Figure 3C). For the cases of the dual crRNAs, ttLbCas12a showed more balanced indel mutation efficiencies between the two gRNAs used in the same construct, especially when the dual crRNA crR1-3.20^ANT1^ with 20-nt gRNAs was examined (Figure 3C, pHRC21, and pHRC22 compared to pHRC15, and pHRC16, respectively). This activity might be one of the reasons that better enhancement of GT efficiency was mediated by ttLbCas12a compared to the WT version.

### ttLbCas12a Shows Better Performance Than WT LbCas12a in Allele-Associated Marker-Free GT at the SlHKT1;2 and SlEPSPS1 Loci

**FIGURE 4:**
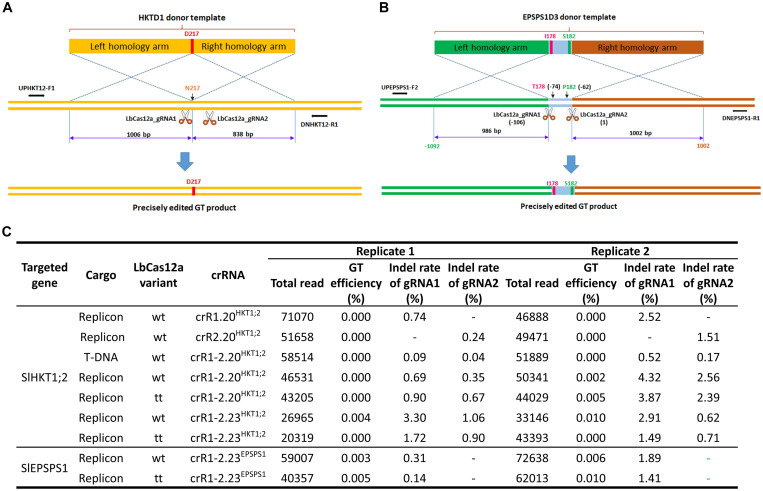
Gene targeting performance of the LbCas12a variants at the SlHKT1;2 and SlEPSPS1 loci. **(A,B)** Schematic diagrams describing the expected GT processes for exchanging the homologous DNA donor template with the genomic sequence at the SlHKT1;2 **(A)** and SlEPSPS1 **(B)** loci. The D217 coding sequence was added during the cloning of the HKTD1 donor for exchange with the N217 sequence of the genomic site. The lengths of homologous arms are shown. Two cutting sites (LbCas12a cutting sites 1 and 2) were planned to support the GT. The reverse and forward primers for amplifying the targeted sites by polymerase chain reaction (PCR) are shown with black arrows. The I178 and S182 coding sequences were added during the cloning of the EPSPS1D3 donor for exchange with the T178 and P182 sequences of the genomic site. The lengths of homologous arms are shown. Two cutting sites (LbCas12a cutting sites 1 and 2) were used for the GT experiments. The reverse and forward primers for amplifying the targeted sites by PCR are shown with black arrows. In panel **(B)**, LbCas12a cutting site 2 is set as position 1, and the other positions are calculated accordingly. The diagrams were drawn not to their actual scales. **(C)** The GT and indel mutation efficiencies were assessed by targeted deep sequencing. At the SlHKT1;2 locus, four different crRNAs (single crRNAs: crR1.20^HKT12^; crR2.20^HKT12^, and dual crRNAs: crR1-2.20^HKT12^; crR1.23^HKT12^) were used for comparison of the LbCas12a variants in GT performance. A T-DNA vector was also used for comparison with the replicon system. With the SlEPSPS1 gene, only one dual crRNA construct (crR1-2.23^EPSPS1^) was used with the two LbCas12a variants. Wt: wild-type LbCas12a; tt: ttLbCas12a.

To further validate and utilize ttLbCas12a in our research on GT, we compared its performance at the two loci without using any donor-associated selection marker, and no allele-associated selection was employed during the experiments. The salt-tolerant allele SlHKT1;2 (N217D) (Figure 4A) was successfully edited using our LbCas12a-based GT system (Vu et al., 2020), although at low efficiency. We introduced two glyphosate-resistant alleles of tomato 5-enolpyruvylshikimate-3-phosphate synthase 1 (SlEPSPS1): (1) T178I and P182S (TIPS allele, corresponding to T103I and P107S in maize, patent no. US6566587B1) (Lebrun et al., 2003) (Figure 4B) and (2) G177A and A268T (GAAT allele, corresponding to G102A and A193T in maize, patent no. US 6225114 B1) (Eichholtz et al., 2001) (Supplementary Figure 9). Extensive comparisons of GT efficiency between LbCas12a and ttLbCas12a were conducted with single crRNAs (crR1.20^HKT1;2^ and crR2.20^HKT1;2^) and dual crRNAs (crR1-2.20^HKT1;2^ and crR1-2.23^HKT1;2^) (Figure 4A, Supplementary Figure 10, and Supplementary Data 1) at the SlHKT1;2 locus. A T-DNA-based GT construct was also used in parallel with the replicon-based construct and the dual crR1-2.20^HKT1;2^ construct. Additional comparisons were performed with the dual crRNAs crR1-2.23^EPSPS1^ and crR1-3.23^EPSPS1^ (Figure 4B, Supplementary Figure 10, and Supplementary Data 1) to replace the TIPS and GAAT alleles, respectively, at the SlEPSPS1 locus.

Gene targeting efficiency calculated by GT deep sequencing reads only showed precise gene editing at both loci in cases of dual crRNAs, although at low efficiencies (Figure 4C). No GT read was obtained from the replicon-based single crRNA constructs and the T-DNA-based dual crRNA regardless of the LbCas12a variants. At the SlHKT1;2 locus, the GT efficiency was higher for ttLbCas12a using the dual 20-nt crRNA (crR1-2.20^HKT1;2^), but contrasting results were obtained with the longer dual crRNA (crR1-2.23^HKT1;2^) (Figure 4C). However, we obtained higher GT efficiency with the 23-nt dual crRNA (crR1-2.23^EPSPS1^) for the replacement of the TIPS allele using ttLbCas12a (Figure 4C). These data indicate that the targeted deep sequencing method may help to identify the GT reads among the constructs, although the read numbers were still too low to be used for statistical comparisons. The indel mutation efficiency obtained from the T-DNA construct was up to 15-fold lower than that of the replicon using the same crR1-2.20^HKT1;2^ (Figure 4C). In this experiment, the indel mutation efficiencies of the single crRNAs were also lower than those of the dual crRNAs. The highest indel mutation activity was obtained with crR1-2.20^HKT1;2^ and LbCas12a, yielding values of 4.32% of LbCas12a_gRNA1 and 2.56% of LbCas12a_gRNA2. The gap between the indel mutation efficiencies of LbCas12a_gRNA1 and LbCas12a_gRNA2 from crR1-2.20^HKT1;2^ was also lower in the case of ttLbCas12a. However, ttLbCas12a did not perform well when it was expressed with the 23-nt dual crRNA (crR1-2.23^HKT1;2^); hence, no GT read was obtained at 10 dpt using the combination (Figure 4C). In general, between the two replicates, higher indel activity in a construct was correlated with higher GT efficiency.

**FIGURE 5:**
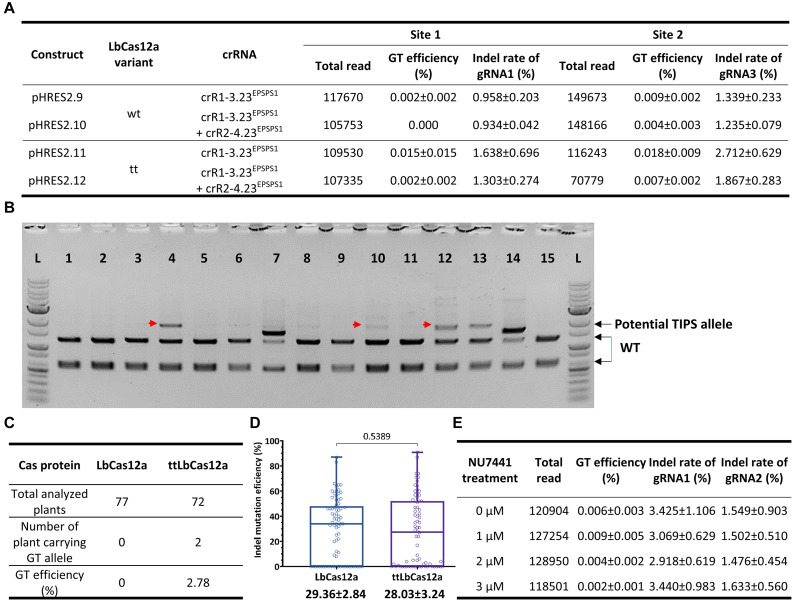
Further assessment of GT performance of the LbCas12a variants at the SlEPSPS1 locus. **(A)** Assessment of GT efficiency by targeted deep sequencing with GT tools using two or four cutting sites with LbCas12a variants at the SlEPSPS1 locus. **(B)** Screening of potential GT events using the CAPS assay at the plant stage. Transformants of each LbCas12a variant were obtained from the transformation of the GT tool with the crR1-2.23^EPSPS1^ expression cassette and used for genomic DNA isolation and PCR amplification of the targeted site with the UPEPSPS1-F2 and DNEPSPS1-R1 primers. The PCR products were purified and digested by the *Bpi*I restriction enzyme since the *Bpi*I site near the targeted site was modified in the DNA donor sequence. The red arrows indicate potential GT bands. 1–15: Representative transformants obtained from the transformation using the GT construct containing LbCas12a and crR1-3.23^EPSPS1^. **(C)** TIPS GT efficiencies obtained with the wt LbCas12a and ttLbCas12a. All the purified PCR products of the transformants that showed undigested bands at WT band size (2,314 bp) in the CAPS assay were analyzed by targeted deep sequencing. Only the transformants carrying more than 5% of the TIPS allele were counted as GT events. **(D)** Boxplots showing indel mutation efficiency induced by LbCas12a and ttLbCas12a. All the purified PCR products used in the CAPS assay were sequenced by the Sanger method, and the ab1 files were subsequently analyzed by ICE Synthego software to reveal the indel mutation and GT efficiencies. The indel mutation efficiencies of all the samples were statistically analyzed using Student’s *t*-test (unpaired, two-tailed, uncorrected) and plotted by GraphPad Prism version 9. The editing efficiencies (mean ± SEM) are shown at the bottom of each box. **(E)** Targeted deep sequencing-mediated evaluation of NU7441 impacts on ttLbCas12a-based GT efficiency.

Additional experiments for the exchange of the GAAT allele using the 23-nt dual crRNAs with two (crR1-3.23^EPSPS1^) or four cleavage sites (crR1-2.23^EPSPS1^ and crR2-4.23^EPSPS1^) and the LbCas12a variants (Supplementary Figures 9, 10) demonstrated that ttLbCas12a performed better in either case. In triplicate, the highest GT efficiency was obtained with ttLbCas12a-expressing constructs (pHRES2.11 compared to pHRES2.9) using the dual crRNA crR1-3.23^EPSPS1^, up to 0.015 ± 0.015% for G177A and 0.018 ± 0.009% for A268T (Figure 5A). Notably, the four crRNA constructs (pHRES2.10 and pHRES2.12) showed a mild reduction in the gRNA1 and gRNA3 indel mutation efficiencies, which were expected to be higher than those of the dual gRNA-expressing constructs due to the synergistic effects of two close cleavage sites (Figure 5A).

To further validate the performance of the LbCas12a variants, we screened and analyzed transformants obtained from the transformation of the LbCas12a- and ttLbCas12a-based constructs for the exchange of the TIPS allele. CAPS screening by *Bpi*I digestion of the PCR products flanking SlEPSPS1-targeted site 1 demonstrated potential TIPS allele-carrying events (Figure 5B). The *Bpi*I site located 84 bp downstream of the T178 codon was modified during the cloning of the homologous DNA donor through the Golden gate cloning method. Therefore, the undigested bands that appeared on the agarose gel were potentially derived from the TIPS-carrying alleles. Using the PCR products of the events that showed undigested bands in the CAPS assay for targeted deep sequencing analysis revealed several transformants carrying the precise GT allele at various frequencies and up to 9.003% in the case of the H281.82 plant generated with the ttLbCas12a construct (Table 1). Interestingly, the GT frequency revealed from ICE Synthego decomposition of the Sanger data of the same event showed only at 7% (Supplementary Figure 11). Two of the transformants (#H281.50 and #H281.82) obtained by the ttLbCas12a-based construct show targeted deep sequencing GT frequency at more than 5% that may be potential for obtaining inherited offsprings (Table 1). Based on that threshold frequency, we revealed that only the ttLbCas12a construct resulted in 2.78% GT efficiency at the plant stage (Figure 5C). Besides, Sanger sequencing of the purified PCR products from 77 and 72 transformants of the wt LbCas12a and ttLbCas12a variant, respectively, and analysis of their.ab1 files by ICE Synthego (Hsiau et al., 2019) identified similar indel mutation efficiencies induced by the Cas proteins (Figure 5D). The average indel mutation efficiency was 29.36 ± 2.84% with LbCas12a and a similar rates for ttLbCas12a (28.03 ± 3.24%) (*p* = 0.5389, Student’s *t*-test). Notably, the SlEPSPS1 TIPS allele-carrying GT plants showed no phenotypic changes compared to the WT plant (Supplementary Figure 12, pHRES2.8 events #82). Several events that contained high indel mutation rates exhibited phenotypic defects compared to WT (Supplementary Figure 12, pHRES2.7 events #27 and 28).

**TABLE 1:** GT efficiency revealed from transformants by targeted deep sequencing.

No.	Cas12a variant	Transformant*	Total reads	GT frequency (%)	Indel rate gRNA1 (%)
1	LbCas12a	H273-4	22620	0.000	99.832
2		H273-10	22730	0.000	100.000
3		H273-12	27245	0.000	100.000
4		H273-13	20569	0.000	100.000
5		H273-27	21295	0.000	99.915
6		H273-28	27632	0.000	99.913
7		H273-29	28425	0.000	100.000
8		H273-30	24552	0.000	99.935
9		H273-37	18258	0.000	99.173
10		H273-44	17520	0.040	80.525
11		H273-46	21303	0.000	100.00
12		H273-49	20361	0.000	99.72
13		H273-54	24244	0.008	99.59
14		H273-81	24823	0.004	100.00
15	ttLbCas12a	H281-2	24049	0.403	95.04
16		H281-3	25966	0.004	100.00
17		H281-12	25973	0.000	99.97
18		H281-19	26908	0.000	100.00
19		H281-26	21048	0.005	99.98
20		H281-27	2823	0.071	93.02
21		H281-36	28424	0.000	99.96
22		H281-42	21637	0.083	18.27
23		H281-48	25078	0.355	13.10
24		H281-49	27841	0.000	99.99
25		H281-50	26571	6.315	82.83
26		H281-53	25272	0.004	45.28
27		H281-55	22867	0.000	99.44
28		H281-60	24409	0.000	98.06
29		H281-82	96066	9.003	6.642

*Samples with undigested bands obtained in the CAPS assays as the potential GT allele-carrying transformants.

### NU7441 Treatment Enhances the ttLbCas12a-Base GT in Tomato

Previously, NU7441 exhibited enhancement of LbCas12a-based GT with SlANT1 as a visible marker (Figure 1B). To further optimize ttLbCas12a-based GT in tomato, we investigated the impacts of various NU7441 concentrations on the GT process using ttLbCas12a constructs with crR1-2.20^HKT12^. Targeted deep sequencing data demonstrated the enhancement of GT efficiency with 1 μM NU7441 added to the culture medium albeit at low read numbers. However, when the NU7441 concentrations were higher, the GT efficiency was lower than that of the mock control (Figure 5E). Notably, the NU7441 treatments at various concentrations showed only mild changes in the indel mutation efficiency of gRNA1 and gRNA2 of the dual crRNA (Figure 5E).

## Discussion

The introduction of DNA DSB(s) at the targeted sites was shown to dramatically enhance GT efficiency in plants (Puchta et al., 1996). DSB repair is dictated by the cNHEJ mechanism due to the abundance of KU70/80 and the other components in the cells (Van Vu et al., 2019). Therefore, the plant HDR efficiency is considerably lower than that of cNHEJ. The HDR pathway is more strictly dependent on the cell cycle, and even during favorable S-G2 phases, HDR must also compete with cNHEJ to repair DNA DSBs. Previously, accumulated data in animal and plant studies showed the possibility of regulating repair pathway determination or inhibiting cNHEJ components by biochemical or chemical approaches (Srivastava et al., 2012; Chu et al., 2015; Maruyama et al., 2015; Robert et al., 2015; Endo et al., 2016; Nishizawa-Yokoi et al., 2016; Song et al., 2016; Zhang et al., 2017). Among the chemicals that exhibited positive effects on HDR or GT in animals, we chose to study several chemicals that inhibit the cNHEJ component(s) to enhance GT efficiency in tomato.

DNA ligase IV was shown to be involved in the last step of cNHEJ-mediated DSB repair to seal the broken ends of DNA DSBs. In a cell-free system, human DNA ligase IV was inhibited by SCR7, a small molecule chemical, by blocking its DNA binding activity (Srivastava et al., 2012). SCR7 at a concentration of 1 μM was shown to enhance CRISPR-Cas9-based GT efficiency up to 5-fold (Chu et al., 2015) or 19-fold (Maruyama et al., 2015) in mammalian and mouse cells, and 10 or 50 μM SCR7 treatment led to reduced transfection efficiency and cell viability. However, our data demonstrate only a moderate increase in GT efficiency with the SpCas9 construct at high levels of SCR7 and no significant enhancement of GT efficiency in the case of LbCas12a (Figure 1A and Supplementary Table 1). Because DNA ligase IV inhibition by SCR7 was irrespective of the DSB configuration (Srivastava et al., 2012), the DNA DSBs generated by either SpCas9 or LbCas12a were not expected to affect the inhibition strength. Nevertheless, we observed contrasted GT efficiency trends between SpCas9 and LbCas12a suggesting that the DSB end configurations differently affected the end ligation by DNA ligase IV. Moreover, since our improved LbCas12a-based GT system showed high efficiency under the experimental conditions, the addition of SCR7, which affects the final step of cNHEJ, may not be recognized easily, and the impacts of its toxicity level may be more visible (Figure 1A). There were limited data regarding the uses of SCR7 for CRISPR-Cas-based GT enhancement in plants; therefore, it is unclear if the SCR7 treatment is species-dependent, since the uptake of SCR7 may be different in plants. The enhancement impacts of SCR7 were also controversial in animals since studies that used human cell lines or rabbit embryos did not show significant improvement of CRISPR-Cas9- or TALEN-based GT efficiency by SCR7 (Gutschner et al., 2016; Song et al., 2016; Yang et al., 2016).

One of the alternative strategies for blocking the cNHEJ pathway is to inhibit the DNA-PKcs or the other DNA-dependent protein kinases of the PI-3K family by NU7441 (Leahy et al., 2004) or KU0060648 (Munck et al., 2012). Studies conducted on mammalian cells showed that CRISPR-Cas9-based GT efficiency is enhanced up to twofold by using NU7441 or KU0060648 (Robert et al., 2015). However, treatment with 1 μM NU7441 and 200 nM KU0060648 did not significantly enhance the SpCas9-based GT efficiency under our experimental conditions, although only NU7441 significantly enhanced the LbCas12a-based GT efficiency up to 1.51-fold in the case of the multireplicon construct (Figure 1B and Supplementary Table 2) thanks to its ability to supply the homologous donor templates by the smaller-sized replicons (Vu et al., 2020). The NU7441 treated sample transformed with the LbCas12a-based single replicon pHR01 showed a higher GT efficiency compared to the control with the *p*-value was closed to 0.05. Since no plant homologs of DNA-PKcs have been identified, these data were surprising but were within our expectations for the existence of other types of plant DNA-dependent protein kinases (Van Vu et al., 2019). More importantly, these data indicate that the inhibitory effects of NU7441 and KU0060648 were selective for the kinase forms and the configurations of DNA DSB ends. Notably, although KU0060648 showed a wider range of inhibition of both DNA-PKcs and PI-3K compared to NU7441, only NU7441 had a positive impact on the LbCas12a GT tools, indicating that the kinase targets of KU006648 and NU7441 in tomato were distinct and that worked differently on the DSB ends formed by SpCas9 and LbCas12a.

To further improve GT systems, recent studies using polyamines (Lee et al., 2019) and silver nitrate, a polyamine biosynthesis regulator, were conducted. In a cell-free assay, polyamines were shown to facilitate RAD51 activities during the formation of synaptic complexes and strand invasion. Depletion of polyamines resulted in the impairment of HDR in mouse hair follicle cells. However, to the best of our knowledge, no direct evidence of the addition of polyamines exerting stimulatory effects on HDR has been reported. Although direct supplementation with polyamines did not affect the CRISPR-Cas-based GT efficiency under our experimental conditions, when we treated the explants with silver nitrate, indirect stimulation of polyamine production using silver nitrate increased GT events, which appeared as purple counting data at 21 dpt (Supplementary Figure 3A). Since the silver nitrate was shown to promote cell proliferation during somatic embryogenesis (Qin et al., 2005; Mythili et al., 2017), it might indirectly stimulate the replication of the geminiviral replicon thereby enhancing the GT efficiency. In the regeneration stage, silver nitrate might also suppress the activities of ethylene released under the stress induced by agrobacteria and tissue culture processes, leading to the promotion of embryogenic callus proliferation and subsequent shoot regeneration (Supplementary Figure 3B) (Roustan et al., 1990; Giridhar et al., 2004).

Previously, we showed that our LbCas12a-based GT system-mediated GT more effectively than did SpCas9 complexes (Vu et al., 2020). However, direct evidence for the comparison is still required, considering that the accessibility of the cleavage sites and loci by CRISPR-Cas complexes may exhibit different results. Therefore, in this study, we selected the well-characterized SlANT1 locus and its two cleavage sites (1 and 3) to compare the two nucleases using the same or very closed gRNA binding sites (Figure 2A and Supplementary Figure 4). The only difference between the SpCas9- and LbCas12a-based vectors was the coding sequence of each of the nucleases, and they were both human-codon optimized (Figure 2B and Supplementary Data 1). As expected, the LbCas12-based GT tools outperformed the SpCas9-based replicons at the same cleavage sites (Figure 2C). Notably, assessment of indel mutation efficiencies of the two systems at the plant stage demonstrated a reverse correlation with the GT efficiencies, as the SpCas9-based indel mutation efficiencies were significantly higher than those of the LbCas12a complexes at the tested cleavage sites (Figure 2D and Supplementary Figure 5). We reason that the strong cleavage activities of the SpCas9 complexes at all the cell cycles may lead to inhibition of the cleavage sites for further recurrent cuts and hence a reduction in the probability of homologous DNA donor-mediated repair by GT in the S-G2 HDR favorable phases. Another important point is that LbCas12a cleaves the targeted sites at a distal side of the TTTV PAM; hence, recurrent cleavages may be possible if the DSB repair of the first cut did not affect the seed sequence located at the proximal side of the PAM (Vu et al., 2020). The view was also supported by a recent study (Huang et al., 2021). We cannot exclude the possibility that the difference in the DNA DSB configurations of SpCas9 (mostly blunt ends) and LbCas12a (cohesive ends) differentially determined repair pathway activation in a spatiotemporal manner, which resulted in the difference in GT efficacy of the two nucleases. There was a different indel mutation observation at the site 1 with the LbCas12a using the 20-nt and 23-nt crRNAs (Figure 2D) since they are supposed to work similarly. However, the *p*-value is not low enough to support a significant difference. In addition, this experiment also indicates that the T-DNA-based gene editing efficiencies were notably low compared to that of the replicon system (Figures 2C,D) at the same cleavage sites due to its low copy nature.

Recently, in attempts to further improve GT efficiency in plants, Puchta’s group found that a single mutation (D156R) of LbCas12a significantly improved GT performance compared to WT nuclease (Merker et al., 2020b; Huang et al., 2021), especially at the optimal temperature for plant growth. However, a direct comparison of the LbCas12a variants in somatic cell systems has not been reported. Our data not only provide a direct comparison of the two nucleases but also showed their crRNA preference (Figure 3) in tomato somatic cells for practical applications. There was no significant improvement in GT efficiency using single crRNAs, but we observed that the ttLbCas12a-based GT might be enhanced with the dual crRNAs although the *p*-value was not less than 0.05 (*p* = 0.0714), especially with the 20-nt crRNAs at the SlANT1 locus (Figure 3C). The weak improvement in GT efficiency mediated by ttLbCas12a compared to the data published by Puchta’s group (Merker et al., 2020a; Huang et al., 2021) could be explained by the high-temperature experimental conditions applied from day 3 to day 12 throughout our study (Supplementary Figure 1), which might reduce the low-temperature tolerance advantages of ttLbCas12a. The slight difference from the 20-nt crRNAs might also be explained by the potential *trans* activities of the LbCas12a-crRNA complexes without affecting the on-target activities (Figure 3C; Fuchs et al., 2019). Further comparison of the LbCas12a variants at the other two loci, SlHKT1;2 and SlEPSPS1, without using a donor or GT allele-associated selection marker showed better performance of ttLbCas12a with dual 20-nt crRNAs at SlHKT12a (Figure 4) and dual 23-nt crRNAs at SlEPSPS1 loci (crR1-2.23^EPSPS1^ and crRNA1-3.23^EPSPS1^) (Figures 4, 5A). The data demonstrated the importance of using two neighboring (Figures 4A,B) or distancing cleavages (Supplementary Figure 9) for efficient GT, as this approach might offer synergistic effects in the case of neighboring cleavages that lead to considerably higher cutting (Chen et al., 2017). In the case of GT alleles that require two distanced sequence modifications, such as the GAAT allele of the SlEPSPS1 loci, two DSBs flanking the targeted sites may ensure simultaneous exchanges of the sequences, since the HDR-based conversion tract generated from each targeted site may not cover the other, due to length limitations (Huang et al., 2021).

Analysis of the transformants obtained from the GT experiment for exchanging the TIPS allele of SlEPSPS1 without using an allele-associated selection marker (i.e., glyphosate herbicide) revealed high GT efficiency (2.78%) at the plant stage only with the ttLbCas12a-based construct (Figure 5C and Table 1). The number of analyzed plants might not be sufficiently high to obtain transformants with more than 5% GT frequency in the case of the LbCas12a-based construct. The transformants carrying up to 9.003% of the GT allele (Table 1) were found with normal phenotypes compared to WT parental plants (Supplementary Figure 12). Nonetheless, it would be important to evaluate the inheritance of the GT allele in the next generations, which was also shown in our previous report with the SlHKT1;2 N217D GT allele using the similar replicon-LbCas12a combination (Vu et al., 2020). We also observed abnormal phenotypes of the transformants carrying high indel mutation rates, especially those obtained from the LbCas12a-based construct. It was due to the high rates of SlEPSPS1 indel mutation alleles present in the same event. The malfunction of the SlEPSPS1 protein might lead to the inefficiency of aromatic amino acid biosynthesis, which causes phenotypic defects. Finally, we confirmed the stimulating impacts of 1 μM NU7441 on ttLbCas12a-based GT performance. However, due to the limited number of the targeted deep sequencing reads that carried the GT allele, even with the thin cotyledon explants, the potentially positive roles of NU7441 in plant GT may be further confirmed by targeted deep sequencing using a protoplast system. Considering that only the ttLbCas12a resulted in events carrying moderate TIPS GT frequencies among fewer than a hundred analyzed plants without using any allele-associated selection marker, ttLbCas12a is a better choice for plant GT. Our findings from this work add more positive information for the improvement of the plant GT system and may further facilitate its practical applications for precision crop breeding.

## Conclusion

The natural HDR efficiency in plant somatic cells is too low to be utilized for practical applications of GT-mediated plant breeding. Continuous efforts to improve GT performance have been undertaken for precision crop breeding. In this study, we further improved the LbCas12a-based GT system with the use of chemical treatments (1 μM of NU7441 and/or AgNO_3_). The impacts of small molecule chemical treatments on GT have not been well studied in crop plants. Therefore, our data for the assessment of the effects of SCR7, NU7441, and KU0060648 treatments on GT efficiency may help to elucidate their impacts and possible targeted components for HDR pathway regulation in plants.

Our data show that LbCas12a outperformed SpCas9 under the same experimental conditions at the SlANT1 loci. Similar effects of the replicon system for GT are also clearly indicated. Despite the milder stimulatory effects on GT performance under our experimental conditions due to the high-temperature protocol, the results of this study indicate that ttLbCas12a is a better choice for future applications in practical GT in plants. Taken together, the combination of the replicon with ttLbCas12a, double cleavages flanking the modification sequence, and the addition of NU7441 and/or AgNO_3_ and appropriate temperature conditions are important parameters for the application of GT in future practical applications in precision plant breeding.

## Experimental Procedures

### System Design for Plant GT in Tomato

The SlANT1, SlHKT1;2, and SlEPSPS1 loci were used to conduct HDR-based DNA insertion and allele replacement experiments. The chemical treatment experiments used a single replicon (pTC217 and pHR01) and multiple-replicon tool (pMR01) vectors from previous works (Cermak et al., 2015; Vu et al., 2020) to target the SlANT1 gene. The SpCas9 containing the pTC217 vector was ordered from Addgene (Plasmid #70018) (Cermak et al., 2015). Further works used the single replicon system as a vector for the delivery of guide RNA and CRISPR-Cas expression cassettes and GT donor templates in this study (Figure 2A and Supplementary Data 1).

For comparison of the SpCas9 and LbCas12a complexes in GT performance, we designed an HDR-mediated insertion of selection markers at the SlANT1 locus that was well studied in our laboratory (Vu et al., 2020). The gRNA binding and cutting sites were selected in a manner that could be used for both SpCas9 (SpCas9_gRNA1 and SpCas9_gRNA2) and LbCas12a (LbCas12a_gRNA1 and LbCas12a_gRNA3) (Figure 2A). We used a single gRNA for each of the GT vectors with either SpCas9 or LbCas12a (Figure 2B). At the cutting site of LbCas12a_gRNA3, two different gRNA lengths (20 and 23 nt) were tested (Figure 2B). All the editing constructs in the experiment were delivered by *Agrobacterium*-mediated transformation of tomato cotyledon explants, and two sets of constructs were cloned: one set with the single geminiviral replicon system (Vu et al., 2020) for the amplification of homologous DNA donors and the other T-DNA set for comparison.

For the assessment and characterization of the activities of ttLbCas12a in GT in comparison with the wild-type version of LbCas12a, similar constructs were designed for targeting the SlANT1 locus with single LbCas12a crRNA expression cassettes. In addition, dual guide RNA expression cassettes combining LbCas12a_gRNA1 (20 and 23 nt) and LbCas12a_gRNA3 (20 and 23 nt) were also used for the GT experiments (Figure 3A and Supplementary Data 1).

A similar system for comparison of ttLbCas12a and wtLbCas12a was designed for targeting the SlHKT1;2 and SlEPSPS1 loci (Figures 4A,B). Single and dual cutting sites were used for SlHKT1;2 (Figure 4A and Supplementary Data 1). The gRNA lengths (20 and 23 nt) were also evaluated at the same locus. For SlEPSPS1, only dual gRNAs 23 nt in length were assessed for GT with the LbCas12a variants (Figure 4B and Supplementary Data 1).

In all the GT vectors, the expression of SpCas9 and LbCas12a variants was driven by a long CaMV 35S promoter that contains an intron (Trp1) at the 5′UTR. A copy of AtUBQ10 intron 1 was also inserted in the coding sequence of the LbCas12a variants (Supplementary Data 1), which was tested previously for GT experiments by Vu et al. (2020). The crRNA and sgRNA expression cassettes were transcribed with the support of the core sequence of the AtU6 promoter (Nekrasov et al., 2013).

### *Agrobacterium*-Mediated Tomato Transformation and Chemical Treatments

*Agrobacterium*-mediated tomato transformation was conducted following our protocol (Supplementary Figure 1) published previously by Vu and coworkers (Vu et al., 2020). For the treatment of chemicals, the chemicals at the tested concentrations were added to the NSEL medium. The 30 μM of silver nitrate was also added to the first subcultured SEL4C medium at the later stage for stimulating somatic embryogenesis. The total treatment time was 5 days [day 3 to day 8 post transformation (dpt)]. For targeted deep sequencing, cotyledon samples were collected at 10 dpt.

### Assessment of GT Efficiency

For assessment of the GT efficiency at SlANT1, purple spot counting was conducted at 21 dpt, and purple plants were recorded at the hardening stage. The GT efficiency reflected by the purple spot numbers was calculated with normalization to the SlANT1 overexpression tool that was transformed in parallel with the GT tool. Our calculation method normalized the number of cells/calli that was edited (and hence carrying p35S-ANT1 expression cassette) by the HDR tools to the number of cells that were successfully transformed with a T-DNA harboring over-expression cassette of the ANT1 gene driven by p35S in the same experimental conditions. This calculation may reflect more actual HDR frequency regarding the editing at the cell levels. A similar calculation was used and accepted elsewhere (Puchta et al., 1993, 1996). This calculation method was also explained in our previous report (Vu et al., 2020).

For assessment of the GT efficiency at the SlHKT1;2 and SlEPSPS1 loci, targeted deep sequencing was conducted with thin cotyledon explants collected at 10 dpt. Sanger sequencing was performed to screen and validate GT plants.

### Targeted Deep Sequencing

Genomic DNAs were isolated from the cotyledon explants (30 explants per sample) or mature leaves (three distinct leaflets from three different young leaves per plant per sample) using the CTAB method. The MiSeq sequencing service (MiniSeq^TM^ System, Illumina, San Diego, CA, United States) was used. MiSeq samples were prepared in three PCRs according to the manufacturer’s guidelines with genomic DNAs as templates for the first PCR. The first and second PCRs used primers listed in Supplementary Table 5, whereas the third PCRs were performed with the manufacturer’s primers to assign sample IDs. The first PCR primers were designed for binding to the upstream and downstream sequences from the homologous donor sequence junctions to avoid amplifying the donor DNA sequences. The second PCR primers were designed to amplify 150–180-bp flanking the targeted base changes at the targeted sites. High-fidelity DNA Taq polymerase (Phusion, NEB, Ipswich, England) was used for PCR. The MiSeq raw data FASTQ files were analyzed by the Cas-Analyzer tool (Park et al., 2016). The indel analysis window was set to 5 bases, with a comparison range covering both read ends. The GT efficiency was assessed using the corresponding donor sequence as the input HDR donor sequence.

### Statistical Analysis

All comparison experiments were conducted with at least three replicates, and data were recorded by purple spot counting, targeted deep sequencing, and plant event screening. Some of the experiments using targeted deep sequencing were conducted in two replicates. The editing data, statistical analysis, and plots were further processed by the MS Excel and GraphPad Prism programs and explained in detail in the legends of figures and/or tables. Pairwise comparison data were tested with Student’s *t*-test with unequal variance and two-tailed parameters. Similar parameters were applied for multiple comparisons using Fisher’s LSD test. A difference was considered to be significant when the statistical tests returned a *p*-value < 0.05.

## Data Availability Statement

The original contributions presented in the study are publicly available. This data can be found here: National Center for Biotechnology Information (NCBI) BioProject database under accession number SUB9867354 and Bioproject number PRJNA738632. The Bioproject can now be accessed at the following link: https://www.ncbi.nlm.nih.gov//bioproject/PRJNA738632.

## Author Contributions

TV and J-YK conceived and designed the research. TV, DD, MT, YSu, and YSo conducted the experiments. TV, DD, MT, and J-YK analyzed the data. TV wrote the manuscript. TV and J-YK finalized the manuscript. All authors read and approved the manuscript.

## Conflict of Interest

The authors declare that the research was conducted in the absence of any commercial or financial relationships that could be construed as a potential conflict of interest.

## Publisher’s Note

All claims expressed in this article are solely those of the authors and do not necessarily represent those of their affiliated organizations, or those of the publisher, the editors and the reviewers. Any product that may be evaluated in this article, or claim that may be made by its manufacturer, is not guaranteed or endorsed by the publisher.

## Supplementary Material

Supplementary Figure 1 *Agrobacterium*-mediated transformation protocol used in this work.

Supplementary Figure 2 Effects of polyamine treatment on the GT efficiency using the multireplicon pMR01.

Supplementary Figure 3 Effects of AgNO_3_ treatment on purple spot numbers and purple shoot regeneration.

Supplementary Figure 4 Map of SpCas9 and LbCas12a binding sites at the SlANT1 locus.

Supplementary Figure 5 Editing performance of the T-DNA and replicon-based SpCas9 and LbCas12a GT tools at various sites of the SlANT1 locus.

Supplementary Figure 6 Representative indel mutation traces of the GT constructs revealed from ICE Synthego analysis.

Supplementary Figure 7 Representative SlANT1 GT events obtained from the study.

Supplementary Figure 8 Thin slice cotyledon explants for the assessment of editing efficiencies by targeted deep sequencing at 10 dpt.

Supplementary Figure 9 Diagram showing the strategy for replacement of two amino acids of the SlEPSPS1 gene.

Supplementary Figure 10 Gene targeting constructs for editing the SlHKT1;2 and SlEPSPS1.

Supplementary Figure 11 ICE Synthego decomposition showing the TIPS allele frequency from the transformant H281.82.

Supplementary Figure 12 Representative transformants obtained using the GT tools for TIPS allele replacement.

Supplementary Table 1 Impacts of SCR7 on GT efficiency.

Supplementary Table 2 Gene targeting performance revealed from the treatment of KU0060648 and NU7441.

Supplementary Table 3 Gene targeting efficiency obtained from polyamine treatment.

Supplementary Table 4 Gene targeting efficiency of the AgNO3 and mock treatments of pMR01.

Supplementary Table 5 Sequences and primers used in this study.

Supplementary Data 1 Sequences used in the study.
