# Supplementary material for: Improvement of the LbCas12a-crRNA System for Efficient Gene Targeting in Tomato
Source: Front Plant Sci. 2021 Aug 10;12:722552. doi: 10.3389/fpls.2021.722552 (PMC8383147; doi:10.3389/fpls.2021.722552)
Supplement: Supplementary Figure 1 — Agrobacterium-mediated transformation protocol used in this work. [file Data_Sheet_1.DOCX]

**Improvement of the LbCas12a-crRNA system for efficient gene targeting in tomato**

Tien Van Vu^1,2,5,*^, Duong Thi Hai Doan^1,5^, Mil Thi Tran^1,3,5^, Yeon Woo Sung^1^, Young Jong Song^1^, Jae-Yean Kim^1,4,*^

^1^Division of Applied Life Science (BK21 FOUR Program), Plant Molecular Biology and Biotechnology Research Center, Gyeongsang National University, Jinju 660-701, Republic of Korea.

^2^National Key Laboratory for Plant Cell Biotechnology, Agricultural Genetics Institute, Km 02, Pham Van Dong Road, Co Nhue 1, Bac Tu Liem, Hanoi 11917, Vietnam.

^3^Crop Science and Rural Development Division, College of Agriculture, Bac Lieu University, Bac Lieu-97000, Vietnam.

^4^Division of Life Science, Gyeongsang National University, 501 Jinju-daero, Jinju 52828, Republic of Korea.

^5^These authors contributed equally as co-first author: Tien Van Vu, Duong Thi Hai Doan, Mil Thi Tran.

*Correspondence: Jae-Yean Kim: [kimjy@gnu.ac.kr](mailto:kimjy@gnu.ac.kr) (ORCID ID: 0000-0002-1180-6232). Tien Van Vu: tienvu.agi@gmail.com (ORCID: 0000-0002-6369-7664).

**SUPPLEMENTAL FIGURES AND TABLES**

**Supplemental Figures and Tables**

| **Supplemental item** | **Page** |
| --- | --- |
| Supplemental Figure 1 *Agrobacterium*-mediated transformation protocol used in this work. | 3 |
| Supplemental Figure 2 Effects of polyamine treatment on the GT efficiency using the multireplicon pMR01. | 4 |
| Supplemental Figure 3 Effects of AgNO3 treatment on purple spot numbers and purple shoot regeneration. | 5 |
| Supplemental Figure 4 Map of SpCas9 and LbCas12a binding sites at the SlANT1 locus. | 6 |
| Supplemental Figure 5 Editing performance of the T-DNA and replicon-based SpCas9 and LbCas12a GT tools at various sites of the SlANT1 locus. | 7 |
| Supplemental Figure 6 Representative indel mutation traces of the GT constructs revealed from ICE Synthego analysis. | 8 |
| Supplemental Figure 7 Representative SlANT1 GT events obtained from the study. | 9 |
| Supplemental Figure 8 Thin slice cotyledon explants for the assessment of editing efficiencies by targeted deep sequencing at 10 dpt. | 10 |
| Supplemental Figure 9 Diagram showing the strategy for replacement of two amino acids of the SlEPSPS1 gene. | 11 |
| Supplemental Figure 10 GT constructs for editing the SlHKT1;2 and SlEPSPS1. | 12 |
| Supplemental Figure 11 ICE Synthego decomposition showing the TIPS allele frequency from the transformant H281.82. | 13 |
| Supplemental Figure 12 Representative GT events obtained using the GT tool for TIPS allele replacement. | 14 |
| Supplemental Table 1. Impacts of SCR7 on GT efficiency. | 15-16 |
| Supplemental Table 2. GT performance revealed from the treatment of KU0060648 and NU7441. | 17 |
| Supplemental Table 3. GT efficiency obtained from polyamine treatment. | 18 |
| Supplemental Table 4. GT efficiency of the AgNO3 and mock treatments of pMR01. | 19 |
| Supplemental Table 5. Sequences and primers used in this study. | 20 |


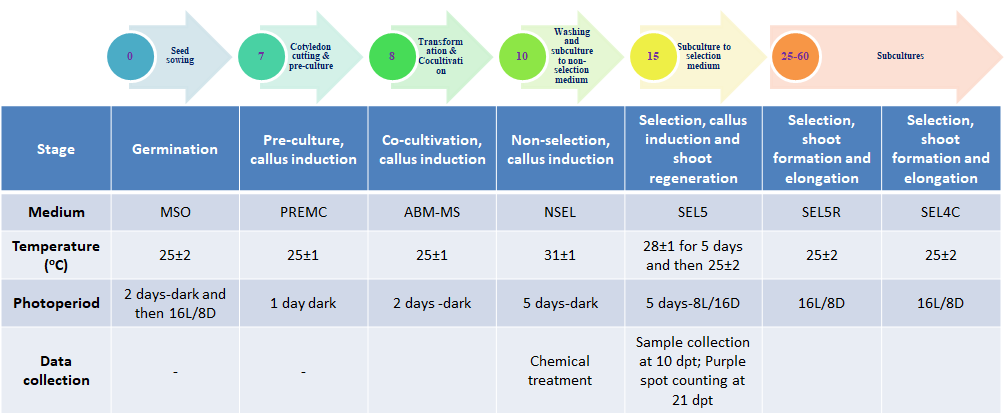


**Supplemental Figure 1 *Agrobacterium*-mediated transformation protocol used in this work.** The step-by-step protocol is presented with each number in the circles indicating the number of days after seed sowing (upper panel), and the treatments used in each step are shown in the lower panel.


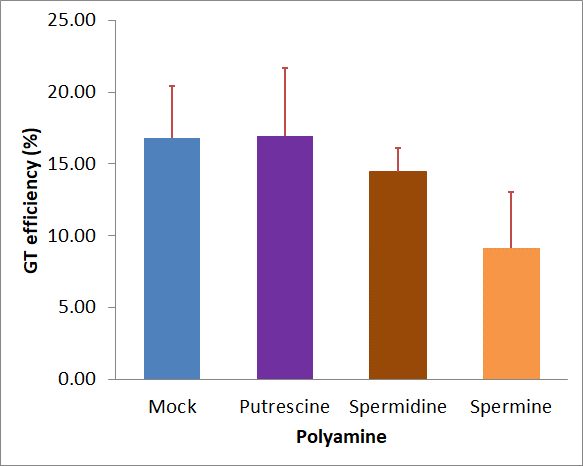


**Supplemental Figure 2 Effects of polyamine treatment on the GT efficiency using the multireplicon pMR01.**


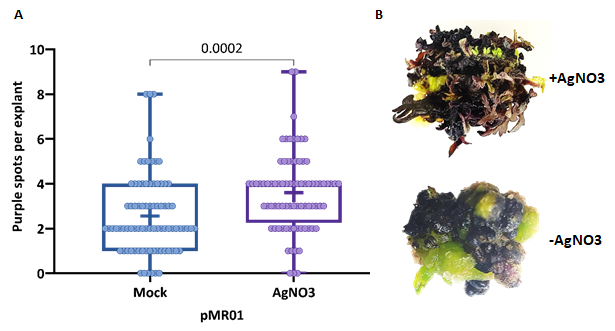


**Supplemental Figure 3 Effects of AgNO3 treatment on purple spot numbers and purple shoot regeneration. A**. Purple spot counts per explants recorded with the AgNO3 treatment at 21 dpt compared to that of the mock control. **B**. A representative of AgNO3-treated purple callus that was regenerating on the culture medium at 45 dpt compared to the mock control.


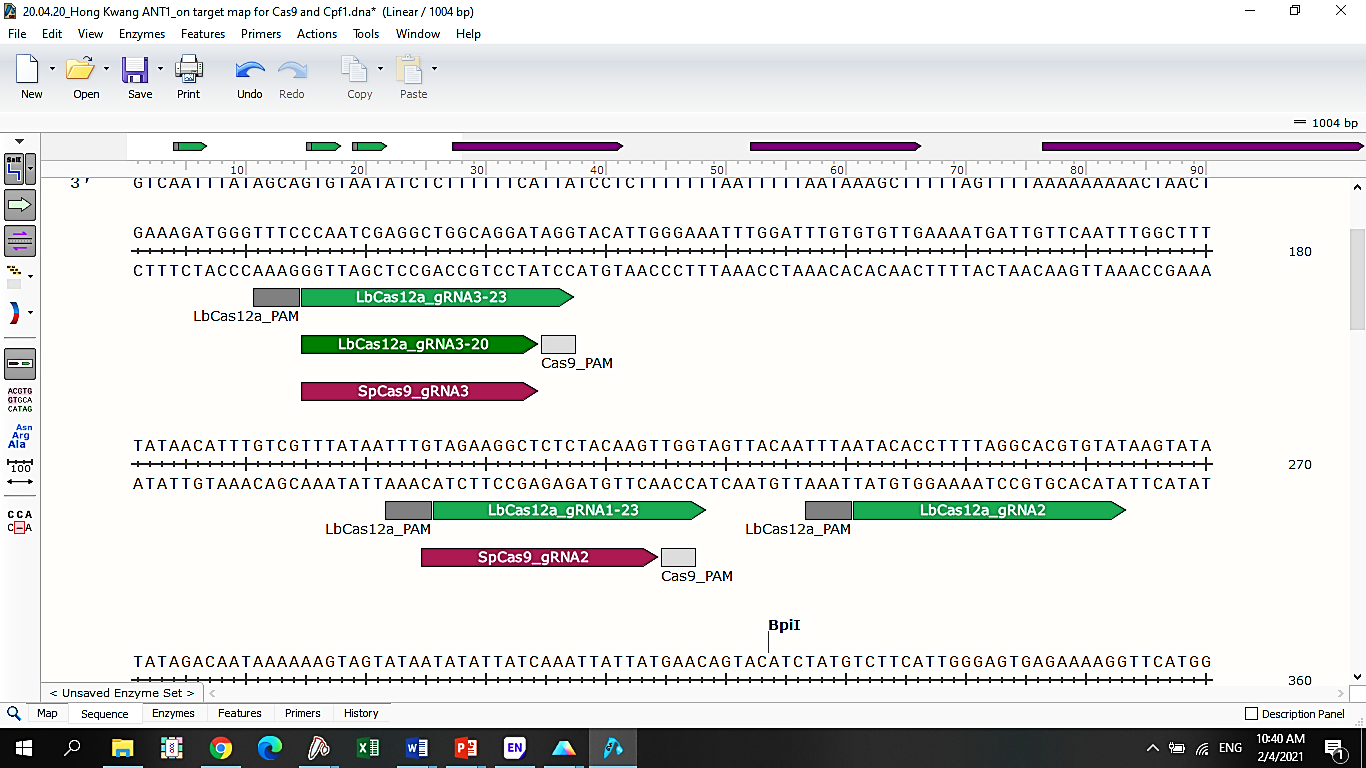


**Supplemental Figure 4 Map of SpCas9 and LbCas12a binding sites at the SlANT1 locus.** Site 1 includes the SpCas9_gRNA2 (20 nt) and LbCas12a_gRNA1 (20 nt), site 2 contains only the LbCas12a_gRNA2 (23-nt) and site 3 is for the SpCas9_gRNA3 (20 nt) and LbCas12a_gRNA3 (20 and 23 nt). The SpCas9_gRNA2 and SpCas9_gRNA3 were used for the sgR2.20^ANT1^ and the sgR3.20^ANT1^, respectively (Supplemental Data S1 file). The LbCas12a_gRNA1 (23 nt), LbCas12a_gRNA21 (23 nt), LbCas12a_gRNA3 (20 nt) and LbCas12a_gRNA3 (23 nt) were used in the crR1.23^ANT1^, crR3.20^ANT1^, crR3.23^ANT1^, crR1-2.23^ANT1^, crR1-3.20^ANT1^ and crR1-3.23^ANT1^ (Supplemental Data S1 file) with the lengths are denoted in the name of the crRNA.


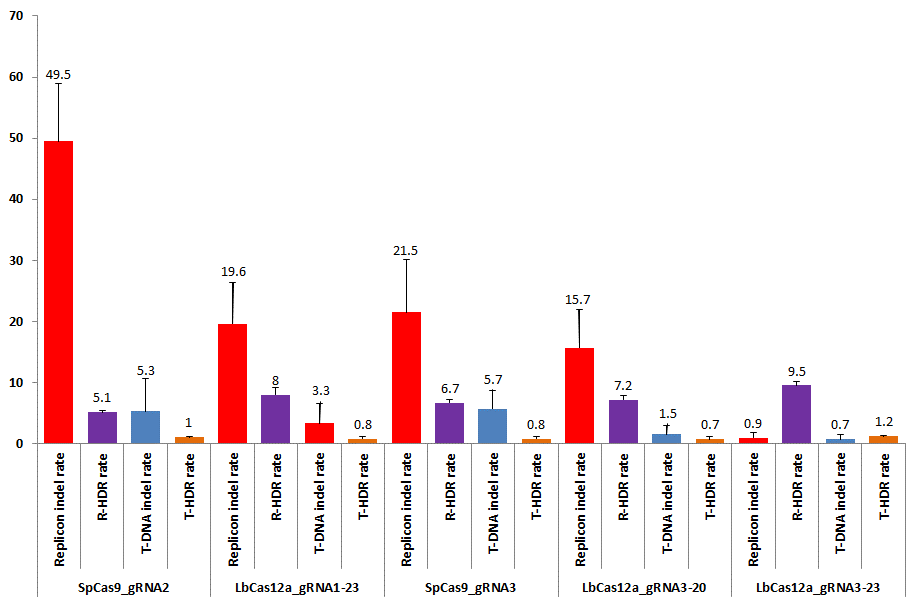


**Supplemental Figure 5 Editing performance of the T-DNA and replicon-based SpCas9 and LbCas12a GT tools at various sites of the SlANT1 locus.** The rates are indicated in %. T-HDR: T-DNA-based HDR; R-HDR: replicon-based HDR.


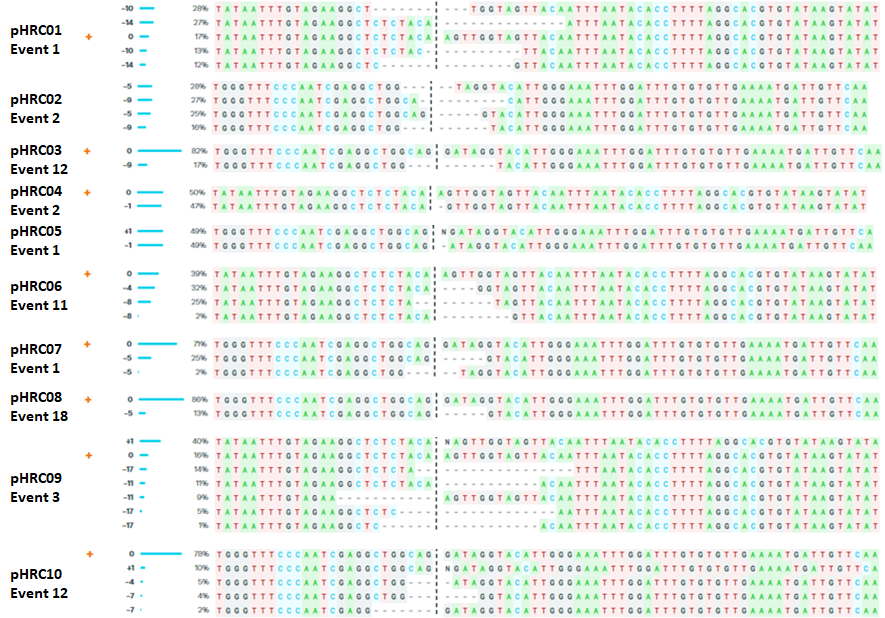


**Supplemental Figure 6 Representative indel mutation traces of the GT constructs revealed from ICE Synthego analysis.**


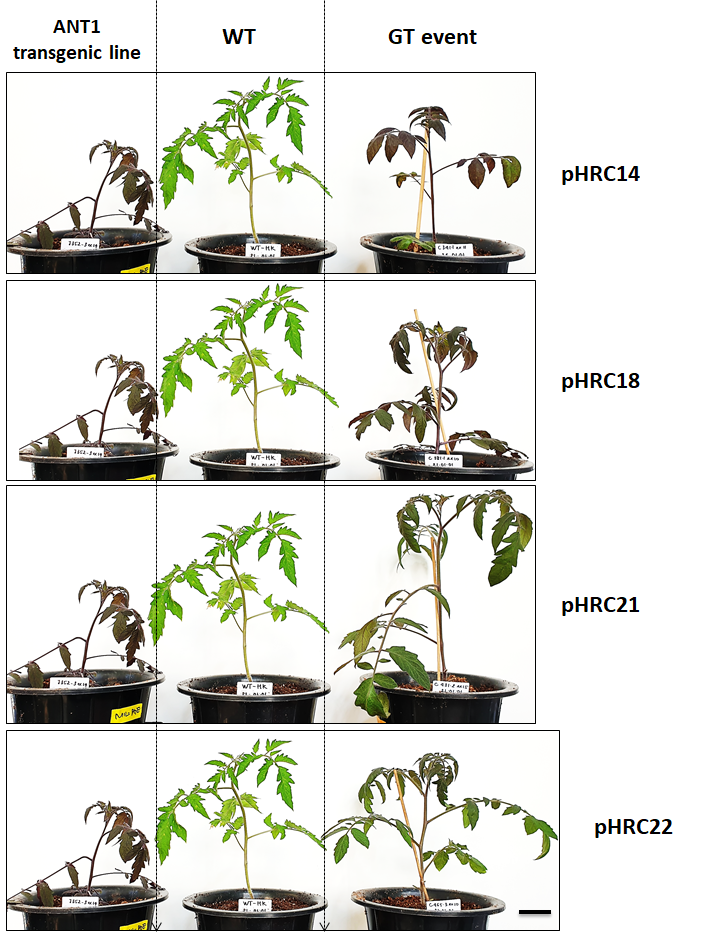


**Supplemental Figure 7 Representative SlANT1 GT events obtained from the study.** The purple phenotype indicates the overexpression of the SlANT1 gene that led to purple color anthocyanin accumulation in the whole plant body of the ANT1 transgenic line or GT events. The WT plant is used as a control. The GT constructs are depicted next to the GT events. Bar=3cm.


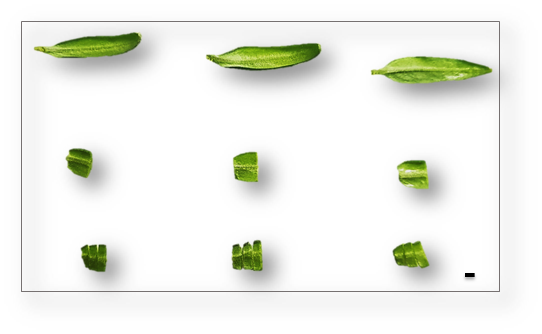


**Supplemental Figure 8 Thin slice cotyledon explants for the assessment of editing efficiencies by targeted deep sequencing at 10 dpt.** The top panel are tomato cotyledons; the middle panel are usual cotyledon explants used in our experiments; the bottom panel shows thin slice cotyledon explants used in our experiments for targeted deep sequencing assessment of editing efficiency at 10 dpt. Bar=1mm.


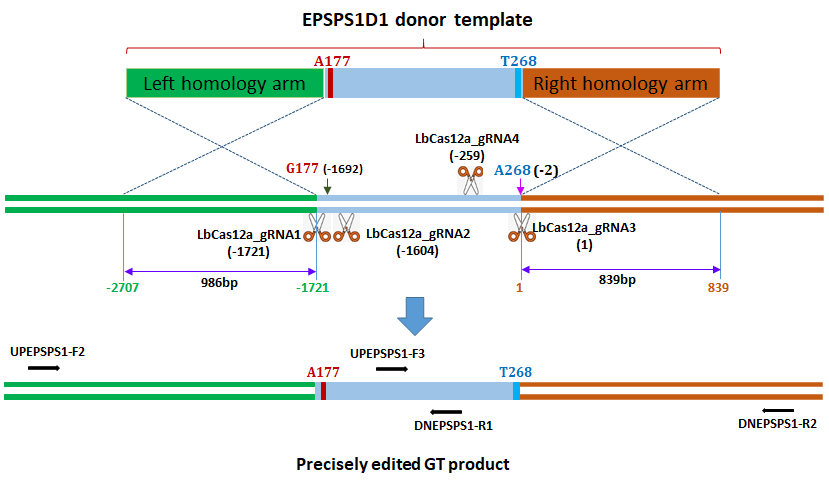


**Supplemental Figure 9 Diagram showing the strategy for replacement of two amino acids of the SlEPSPS1 gene.** The A177 and T268 coding sequences were added during the cloning of the EPSPS1D1 donor for exchanging with the G177 and A268 sequences of the genomic site. The lengths of homologous arms are shown. Two cutting sites (LbCas12a cutting sites 1 and 3) were used for the GT experiments. The reverse and forward primers for amplifying the targeted sites by PCRs are shown with black arrows. The LbCas12a cutting site 3 is set as position 1 and the other positions are calculated accordingly. The diagrams were drawn not to their actual scales.


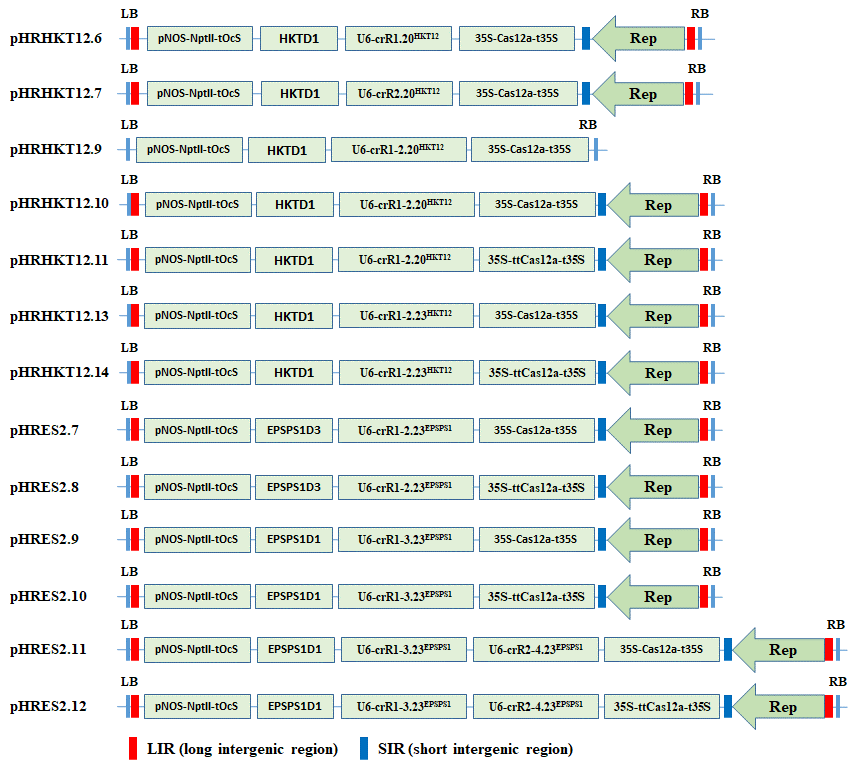


**Supplemental Figure 10 GT constructs for editing the SlHKT1;2 and SlEPSPS1.**


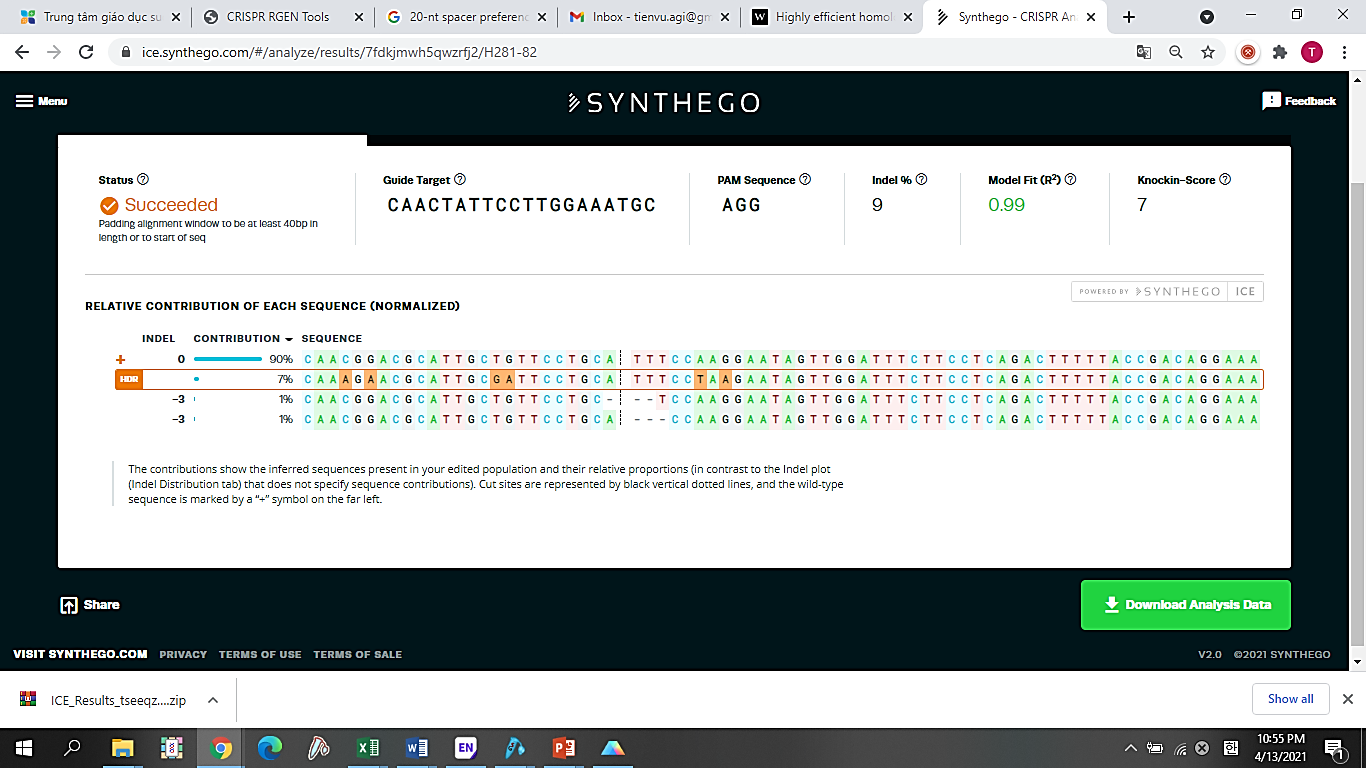


**Supplemental Figure 11 ICE Synthego decomposition showing the TIPS allele frequency from the transformant H281.82.**


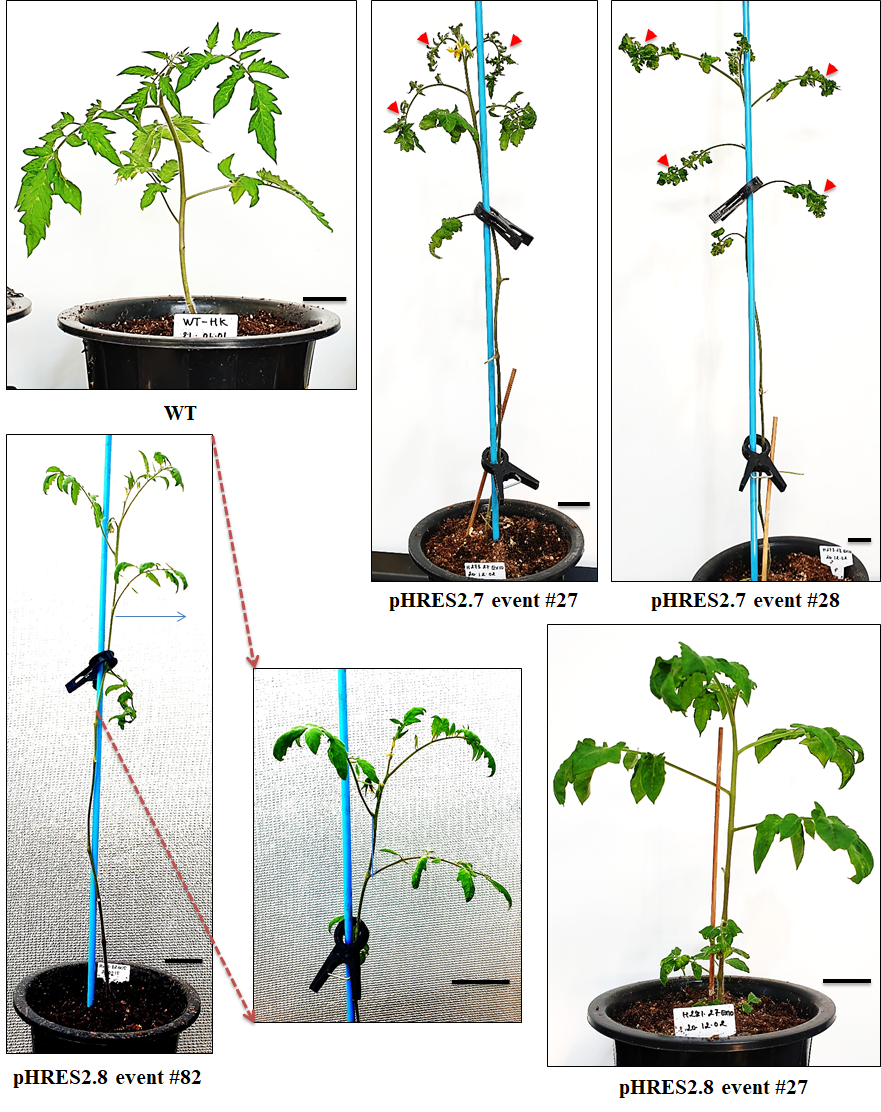


**Supplemental Figure 12 Representative transformants obtained using the GT tools for TIPS allele replacement.** The LbCas12a (pHRES2.7 GT tool)- and ttLbCas12a (pHRES2.8 GT tool)-based GT events are numbered at the bottom of each panel. The WT plant is shown for phenotypic comparison. The red arrows point to the abnormal leaves. Bars=3cm.

**Supplemental Table 1. Impacts of SCR7 on GT efficiency.**

| **Construct** | **SCR7 concentration (μM)** | **GT efficiency (%)** | | | **Average (%)** | **SEM** | **p value (t.test of treatment vs. mock)** |
| --- | --- | --- | --- | --- | --- | --- | --- |
|  |  | Replicate 1 | Replicate 2 | Replicate 3 |  |  |  |
| pTC217 | 0 | 2.09 | 3.02 | 2.26 | 2.46 | 0.29 | - |
|  | 1 | 1.84 | 1.44 | 2.13 | 1.80 | 0.20 | 0.1460 |
|  | 10 | 3.27 | 3.37 | 3.21 | 3.28 | 0.05 | 0.0760 |
|  | 50 | 2.65 | 4.15 | 3.00 | 3.27 | 0.45 | 0.0810 |
| pHR01 | 0 | 5.70 | 6.43 | 8.72 | 6.95 | 0.91 | - |
|  | 1 | 4.52 | 5.78 | 7.36 | 5.89 | 0.82 | 0.4432 |
|  | 10 | 3.87 | 7.29 | 7.82 | 6.33 | 1.24 | 0.6490 |
|  | 50 | 2.93 | 4.27 | 5.22 | 4.14 | 0.66 | 0.0656 |

**Supplemental Table 2. GT performance revealed from the treatment of KU0060648 and NU7441.**

| **Construct** | **Chemical treatment** | **GT efficiency (%)** | | | **Average (%)** | **SEM** | **p value (t.test of treatment vs. mock)** |
| --- | --- | --- | --- | --- | --- | --- | --- |
|  |  | **Replicate 1** | **Replicate 2** | **Replicate 3** |  |  |  |
| pTC217 | Mock | 5.76 | 5.22 | 3.96 | 4.98 | 0.53 | - |
|  | KU0060648 | 12.32 | 6.05 | 5.32 | 7.90 | 2.22 | 0.1981 |
|  | NU7441 | 5.61 | 6.30 | 8.70 | 6.87 | 0.94 | 0.3847 |
| pHR01 | Mock | 3.84 | 8.99 | 8.69 | 7.17 | 1.67 | - |
|  | KU0060648 | 6.70 | 8.64 | 7.63 | 7.66 | 0.58 | 0.8067 |
|  | NU7441 | 9.32 | 14.06 | 9.83 | 11.07 | 1.50 | 0.0848 |
| pMR01 | Mock | 10.40 | 9.78 | 12.84 | 11.01 | 0.94 | - |
|  | KU0060648 | 10.43 | 10.06 | 12.91 | 11.13 | 0.89 | 0.9315 |
|  | NU7441 | 14.49 | 18.17 | 15.00 | 15.88 | 1.15 | 0.0136 |

**Supplemental Table 3. GT efficiency obtained from polyamine treatment.**

| **Treatment** | **GT efficiency (%)** | | | **SD** | **SEM** |
| --- | --- | --- | --- | --- | --- |
|  | **Rep.1** | **Rep.2** | **Average** |  |  |
| Mock | 20.4 | 13.2 | 16.78 | 5.11 | 3.62 |
| Putrescine | 21.7 | 12.1 | 16.93 | 6.76 | 4.78 |
| Spermidine | 16.1 | 12.8 | 14.44 | 2.34 | 1.66 |
| Spermine | 13.1 | 5.2 | 9.14 | 5.57 | 3.94 |

**Supplemental Table 4. GT efficiency of the AgNO3 and mock treatments of pMR01.**

| **Treatment** | **GT efficiency (%)** | | | **SD** | **SEM** |
| --- | --- | --- | --- | --- | --- |
|  | Replicate 1 | Replicate 2 | Average |  |  |
| Mock | 12.9 | 11.0 | 11.96 | 1.37 | 0.97 |
| AgNO3 | 15.6 | 14.2 | 14.90 | 1.06 | 0.75 |

**Supplemental Table 5. Sequences and primers used in this study.**

| **No.** | **Name** | | **Sequence (5’-3’)** | **Note** |
| --- | --- | --- | --- | --- |
| **gRNA and donor sequences** | | | | |
| 1 | gR1.20^ANT1^ | | TAGAAGGCTCTCTACAAGTT | SlANT1 LbCas12a_gRNA1, 20 nt |
| 2 | gR1.23^ANT1^ | | TAGAAGGCTCTCTACAAGTTGGT | SlANT1 LbCas12a_gRNA1, 23 nt |
| 3 | gR3.20^ANT1^ | | CCAATCGAGGCTGGCAGGAT | SlANT1 LbCas12a_gRNA3, 20 nt |
| 4 | gR3.23^ANT1^ | | CCAATCGAGGCTGGCAGGATAGG | SlANT1 LbCas12a_gRNA3, 23 nt |
| 5 | sgR2^ANT1^ | | GTAGAAGGCTCTCTACAAGT | SlANT1 SpCas9_gRNA2, 20nt |
| 6 | sgR3^ANT1^ | | CCAATCGAGGCTGGCAGGAT | SlANT1 SpCas9_gRNA3, 20nt |
| 7 | gR1.20^HKT12^ | | ACTATTCACCACAGTATCAA | SlHKT1;2 LbCpf1_gRNA1, 20nt |
| 8 | gR1.23^HKT12^ | | ACTATTCACCACAGTATCAACTT | SlHKT1;2 LbCas12a_gRNA1, 23nt |
| 9 | gR2.20^HKT12^ | | CCTACAAATGAAAACATGAT | SlHKT1;2 LbCas12a_gRNA2, 20nt |
| 10 | gR2.23^HKT12^ | | CCTACAAATGAAAACATGATGAT | SlHKT1;2 LbCas12a_gRNA2, 23nt |
| 11 | gR1.23^EPSPS1^ | | CAAGGAATAGTTGGATTTCTTC | SlEPSPS1 LbCas12a_gRNA1, 23nt |
| 12 | gR2.23^EPSPS1^ | | AATCGTTCCTTCTTCGTGCCAT | SlEPSPS1 LbCas12a_gRNA2, 23nt |
| 13 | gR3.23^EPSPS1^ | | AATCTCCACATCTCCTAGAGCCA | SlEPSPS1 LbCas12a_gRNA3, 23nt |
| 14 | gR4.23^EPSPS1^ | | CTCCAAAAGTCTCTTGTCCCTCC | SlEPSPS1 LbCas12a_gRNA4, 23nt |
| **Primers for amplification of sequences flanking targeted sites** | | | | |
| 15 | UPANT1-F1 | | TGCGATGATCTACGGTAACAAA | SlANT1 left junction, 1485 bp |
| 16 | NPTII-R1 | | GCGTGCAATCCATCTTGTTC |  |
| 17 | ZY010F | | ACGTAAGGGATGACGCACA | SlANT1 right junction, 1380 bp |
| 18 | TC140R | | TACCACCGGTCCATTCCCTA |  |
| 19 | UPANT1-F1 | | TGCGATGATCTACGGTAACAAA | SlANT1 full targeted site, 4707 bp |
| 20 | TC140R | | TACCACCGGTCCATTCCCTA |  |
| 21 | TC140F | | GGAAAATGGCATCTTGTTCCC | SlANT1 control, 1056 bp |
| 22 | TC140R | | TACCACCGGTCCATTCCCTA |  |
| 23 | GR-F1 | | TTGAGATGAGCACTTGGGATAG | Replicon, 557 bp |
| 24 | pCf.ANT1-R4 | | ACCTCAACGACGCAAGTATT |  |
| 25 | RB-qF2 | | CTCTTAGGTTTACCCGCCAATA | T-DNA, 961 bp |
| 26 | RRA-R6 | | GTTCAGGTTGTGGAGGGAATAA |  |
| 27 | UPHKT12-F1 | | TTCACATGCTTTGACCCATAAA | SlHKT1;2, 2034 bp |
| 28 | DNHKT12-R1 | | CTCTTCCTATAAACGTGCACTCA |  |
| 29 | HKT12-sF1 | | CAAAGATTATGAGCTAGGGAATGT | SlHKT1;2 targeted site sequencing |
| 20 | GR-F1 | | TTGAGATGAGCACTTGGGATAG | SlHKT1;2 replicon, 483 bp |
| 21 | pNOS-cR1 | | AACGTGACTCCCTTAATTCTCC |  |
| 22 | RB-qF2 | | CTCTTAGGTTTACCCGCCAATA | SlHKT1;2 T-DNA, 2236 bp |
| 23 | LbCpf1-cR1 | | CGAGAAGCTGGATAAGGTGAA |  |
| 24 | GAPDH-F1 | | CCATAACCTAATTTCTCTCTC | SlGAPDH, 1073 bp |
| 25 | GAPDH-R1 | | GTCATGAGACCCTCAACAAT |  |
| 26 | UPEPSPS1-F2 | | ACATGTAAGTTAGACAAGAGCTAGG | SlEPSPS1 targeted site 1, 2314 bp;  targeted site 3, 3910 bp |
| 27 | DNEPSPS1-R1 | | GGGAGTGAGTGCATACTTGTT |  |
| 28 | DNEPSPS1-R2 | | GGGTAGCATGACAAAGGACATC |  |
| 29 | EPSPS1-sF3 | | GGAGATATTTGTTAGAGTACCATCA | SlEPSPS1 targeted site 1 sequencing |
| 30 | EPSPS1-sR1 | | TAGCTAGCACTTGAGGCATCTC | SlEPSPS1 targeted site 3 sequencing |
| **Primers for miseq sample preparation** | | | | |
| 31 | | ANNG-F1 | CCCTCTCACGATTAATGATAGTT | First PCR for SlANT1 |
| 32 | | ANNG-R1 | CCAGCTCTTATGGGAACAAG |  |
| 33 | | ANNG-F2 | ACACTCTTTCCCTACACGACGCTCTTCCGATCTGCAGGATAGGTACATTGGGA | First PCR for SlANT1 site 1 |
| 34 | | ANNG-R2 | GTGACTGGAGTTCAGACGTGTGCTCTTCCGATCT GTCTATATATACTTATACACGTGCCTAAAAGG |  |
| 35 | | ANNG-F3 | ACACTCTTTCCCTACACGACGCTCTTCCGATCTCGTCACATTATAGAGAAA | First PCR for SlANT1 site 3 |
| 36 | | ANNG-R3 | GTGACTGGAGTTCAGACGTGTGCTCTTCCGATCT GCCAAATTGAACAATCATTTTCAACACAC |  |
| 37 | | UPHKT12-F1 | TTCACATGCTTTGACCCATAAA | First PCR for SlHKT1;2 |
| 38 | | DNHKT12-R1 | CTCTTCCTATAAACGTGCACTCA |  |
| 39 | | HKPE-F2 | ACACTCTTTCCCTACACGACGCTCTTCCGATCTCGCCAAACAAATCCTTGACC | Second PCR for SlHKT1;2 |
| 40 | | HKPE-R2 | GTGACTGGAGTTCAGACGTGTGCTCTTCCGATCTTGAGGGATAAGAATGAGAAGAAGAC |  |
| 41 | UPEPSPS1-F2 | | ACATGTAAGTTAGACAAGAGCTAGG | First PCR for SlEPSPS1 site 1 |
| 42 | DNEPSPS1-R1 | | GGGAGTGAGTGCATACTTGTT |  |
| 43 | EPPE-F2 | | ACACTCTTTCCCTACACGACGCTCTTCCGATCTGGCAGTTTCCTGTCGGTAA | First PCR for SlEPSPS1 site 1 |
| 44 | EPPE-R2 | | GTGACTGGAGTTCAGACGTGTGCTCTTCCGATCTAAACAAATGGCACGAAGAAGG |  |
| 45 | UPEPSPS1-F2 | | ACATGTAAGTTAGACAAGAGCTAGG | First PCR for SlEPSPS1 site 3 |
| 46 | DNEPSPS1-R2 | | GGGTAGCATGACAAAGGACATC |  |
| 47 | SlEPSPS1-F2.2 | | ACACTCTTTCCCTACACGACGCTCTTCCGATCTCATATCTATCAGGTAAAGCTCTCTGG | Second PCR for SlEPSPS1 site 3 |
| 48 | SlEPSPS1-R2.2 | | GTGACTGGAGTTCAGACGTGTGCTCTTCCGATCTCGCTCCATCAACTTCAGTGTCATTTC |  |
