# Supplementary material for: Improvement of the LbCas12a-crRNA System for Efficient Gene Targeting in Tomato
Source: Front Plant Sci. 2021 Aug 10;12:722552. doi: 10.3389/fpls.2021.722552 (PMC8383147; doi:10.3389/fpls.2021.722552)
Supplement: Supplementary Data 1 — Sequences used in the study. [file Data_Sheet_2.PDF]

SEQUENCES USED IN THE STUDY

crRNA expression cassettes and donor sequences for SlANT1 locus

- U6-crR1.20^{ANT1} expression cassette:

TGATCAAAAGTCCCACATCGATCAGGTGATATATAGCAGCTTAGTTTATATAATGATAGAGTCGACATAGCGA
TTGtAATTTCTACTAAGTGTAGATTAGAAGGCTCTCTACAAGTTTTTTTTT
Red font: AtU6 promoter; green font: LbCas12a scaffold; light blue font: LbCas12a_gRNA1, 20nt; black G: transcription start; black TTTTTT: termination sequence.

- U6-crR1.23^{ANT1} expression cassette:

TGATCAAAAGTCCCACATCGATCAGGTGATATATAGCAGCTTAGTTTATATAATGATAGAGTCGACATAGCGA
TTGtAATTTCTACTAAGTGTAGATTAGAAGGCTCTCTACAAGTTGGTTTTTTTT
Red font: AtU6 promoter; green font: LbCas12a scaffold; blue font: LbCas12a_gRNA1, 23nt; black G: transcription start; black TTTTTT: termination sequence.

- U6-crR3.20^{ANT1} expression cassette:

TGATCAAAAGTCCCACATCGATCAGGTGATATATAGCAGCTTAGTTTATATAATGATAGAGTCGACATAGCGA
TTGtAATTTCTACTAAGTGTAGATCCAATCGAGGCTGGCAGGATTTTTTTT
Red font: AtU6 promoter; green font: LbCas12a scaffold; purple font: LbCas12a_gRNA3, 20nt; black G: transcription start; black TTTTTT: termination sequence.

- U6-crR3.23^{ANT1} expression cassette:

TGATCAAAAGTCCCACATCGATCAGGTGATATATAGCAGCTTAGTTTATATAATGATAGAGTCGACATAGCGA
TTGtAATTTCTACTAAGTGTAGATCCAATCGAGGCTGGCAGGATAGGTTTTTTT
Red font: AtU6 promoter; green font: LbCas12a scaffold; orange font: LbCas12a_gRNA3, 23nt; black G: transcription start; black TTTTTT: termination sequence.

- Dual U6-crR1-3.20^{ANT1} expression cassette:

TGATCAAAAGTCCCACATCGATCAGGTGATATATAGCAGCTTAGTTTATATAATGATAGAGTCGACATAGCGA
TTGtAATTTCTACTAAGTGTAGATTAGAAGGCTCTCTACAAGTTTAATTTCTACTAAGTGTAGATCCAATCGAG
GCTGGCAGGATTTTTTT
Red font: AtU6 promoter; green font: LbCas12a scaffolds; light blue font: LbCas12a_gRNA1, 20nt; purple font: LbCas12a_gRNA3, 20nt; black G: transcription start; black TTTTTT: termination sequence.

- Dual U6-crR1-3.23^{ANT1} expression cassette:

TGATCAAAAGTCCCACATCGATCAGGTGATATATAGCAGCTTAGTTTATATAATGATAGAGTCGACATAGCGA
TTGtAATTTCTACTAAGTGTAGATTAGAAGGCTCTCTACAAGTTGGTTAATTTCTACTAAGTGTAGATCCAATC
GAGGCTGGCAGGATAGGTTTTTT
Red font: AtU6 promoter; green font: LbCas12a scaffolds; blue font: LbCas12a_gRNA1, 23nt; orange font: LbCas12a_gRNA3, 23nt; black G: transcription start; black TTTTTT: termination sequence.

- U6-sgR2^{ANT1} expression cassette:

TGATCAAAAGTCCCACATCGATCAGGTGATATATAGCAGCTTAGTTTATATAATGATAGAGTCGACATAGCGA
TTGGTAGAAGGCTCTCTACAAGTgttttagagctagaaatagcaagttaaaataaggctagtccgttatcaacttgaaaaagtggcac
cgagtcggtgcTTTTTTT
Red font: AtU6 promoter; green font: SpCas9 scaffold; purple font: SpCas9_gRNA2, 20nt; black G: transcription start; black TTTTTT: termination sequence.

- U6-sgR3^{ANT1} expression cassette:

TGATCAAAAGTCCCACATCGATCAGGTGATATATAGCAGCTTAGTTTATATAATGATAGAGTCGACATAGCGA
TTGCCAATCGAGGCTGGCAGGATgttttagagctagaaatagcaagttaaaataaggctagtccgttatcaacttgaaaaagtggca
ccgagtcggtgcTTTTTTT
Red font: AtU6 promoter; green font: SpCas9 scaffold; blue font: SpCas9_gRNA3, 20nt; black G: transcription start; black TTTTTT: termination sequence.

- ANT1D2 donor sequence

TTGGTCCCCAAGTACTTAAATTGGTCCCCAAGTACTTACCACAACACTTGTCGGTGAGATTATTTAATGCTGAT
TAGATTAGACAAAAATTAATTAGTTTTGAGTAGTGGCGTAAGTGTAAATAATTAGTCTCTTTTTTAACTTAGAA
AATAGTTTAATCCTTAGTATAAATAGTCAAAATCACTGGAATGAAAAACAGTTTTTAATTTTTCCAAATTTGATT
CTGATACCATGTTAAATTCGTGGTTCAAAATCACTGCAATGAAAAGAGCAATATTGTTTAACTTTTTTTAGGAA
AATCGAATTGATTTATAGTCAGTTGATATAGAGTGAATACATAAGGAACATATACAGTTGATACAATTGTATA
ATTCGTTCATACACTTAATACAAAGTGAACCCACAAGGAACATATACACTTAATATAATTGTATTCCTTGATAC
AAACCAATTTTGTTCGTGTCTCTACTCTCTATTTCAATTTCGCTTGACTCTTTACTTTTTCTAATATGTAGCTATAA
ATCGTAATTAAACAATACTATATCTCTAAATCTCTTATTAAGCTCAAACTATGGTCATATTCGAAAAAATCCTTT
TAAATATTGGTCCCCTCTCACGATTAATGATAGTTATAACTAACATTCAAATTTTAGTTGTACTTGACATCTAAA
ACTTAAAAAATAGTACAAGTTAACTTTTTCTTTTTTTTAAAAAAAGGAAATACTTGTATTTATTTTTTTAATATAT
AGTTATATTTTTGGTTATTTGAAAATACTTGATCTGTCATGTATGCTCAGTTAAATATCGTCACATTATAGAGAA
AAAAGTAATAGGAGAAAAAAATTAAAAATTATTTCGAAAAATCAAAATTTTTTTTTGATTGAAATGAAAGATG
GGTTTCCCAATCGAGGCTGGCAGGATAGGTACATTGGGAAATTTGGATTTGTGTGTTGAAAATGATTGTTCAA
TTTGGCTTTTATAACATTTGTCGTTTATAAGGTGTAGAAGGCTCTCTACAAGTTGGTAGTTACAATTTAATACA
CCTGAATTCGGATCCGGAGCGGAGAATTAAGGGAGTCACGTTATGACCCCCGCCGATGACGCGGGACAAGC
CGTTTTACGTTTGGAACTGACAGAACCGCAACGTTGAAGGAGCCACTGAGCCGCGGGTTTCTGGAGTTTAAT
GAGCTAAGCACATACGTCAGAAACCATTATTGCGCGTTCAAAAGTCGCCTAAGGTCACTATCAGCTAGCAAAT
ATTTCTTGTCAAAAATGCTCCACTGACGTTCCATAAATTCCCCTCGGTATCCAATTAGAGTCTCATATTCACTCT
CCTATTTTTACAACAATTACCAACAACAACAAACAACAAACAACATTACAATTACATTTACAATTACCATGGTT
GAACAAGATGGATTGCACGCAGGTTCTCCGGCCGCTTGGGTGGAGAGGCTATTCGGCTATGACTGGGCACAA
CAGACAATCGGCTGCTCTGATGCCGCCGTGTTCCGGCTGTCAGCGCAGGGGCGCCCGGTTCTTTTTGTCAAGA
CCGACCTGTCCGGTGCCCTGAATGAACTGCAGGACGAGGCAGCGCGGCTATCGTGGCTGGCCACGACGGGC
GTTCCTTGCGCAGCTGTGCTCGACGTTGTCACTGAAGCGGGAAGGGACTGGCTGCTATTGGGCGAAGTGCCG
GGGCAGGATCTCCTGTCATCTCACCTTGCTCCTGCCGAGAAAGTATCCATCATGGCTGATGCAATGCGGCGGC
TGCATACGCTTGATCCGGCTACCTGCCCATTCGACCACCAAGCGAAACATCGCATCGAGCGAGCACGTACTCG
GATGGAAGCCGGTCTTGTCGATCAGGATGATCTGGACGAAGAGCATCAGGGGCTCGCGCCAGCCGAACTGT
TCGCCAGGCTCAAGGCGCGCATGCCCGACGGCGAGGATCTCGTCGTGACTCATGGCGATGCCTGCTTGCCGA
ATATCATGGTGGAAAATGGCCGCTTTTCTGGATTCATCGACTGTGGCCGGCTGGGTGTGGCGGACCGCTATC
AGGACATAGCGTTGGCTACCCGTGATATTGCTGAAGAGCTTGGCGGCGAATGGGCTGACCGCTTCCTCGTGC
TTTACGGTATCGCCGCTCCCGATTCGCAGCGCATCGCCTTCTATCGCCTTCTTGACGAGTTCTTCTGAGCGGGA
CTCTGGGGTTCGCTAGAGTCCTGCTTTAATGAGATATGCGAGACGCCTATGATCGCATGATATTTGCTTTCAAT
TCTGTTGTGCACGTTGTAAAAAACCTGAGCATGTGTAGCTCAGATCCTTACCGCCGGTTTCGGTTCATTCTAAT
GAATATATCACCCGTTACTATCGTATTTTTATGAATAATATTCTCCGTTCAATTTACTGATTGTACCCTACTACTT
ATATGTACAATATTAAAATGAAAACAATATATTGTGCTGAATAGGTTTATAGCGACATCTATGATAGAGCGCC

ACAATAACAAACAATTGCGTTTTATTATTACAAATCCAATTTTAAAAAAAGCGGCAGAACCGGTCAAACCTAA
AAGACTGATTACATAAATCTTATTCAAATTTCAAAAGTGCCCCAGGGGCTAGTATCTACGACACACCGAGCGG
CGAACTAATAACGCTCACTGAAGGGAACTCCGGTTCCCCGCCGGCGCGCATGGGTGAGATTCCTTGAAGTTG
AGTATTGGCCGTCCGCTCTACCGAAAGTTACGGGCACCATTCAACCCGGTCCAGCACGGCGGCCGGGTAACC
GACTTGCTGCCCCGAGAATTATGCAGCATTTTTTTGGTGTATGTGGGCCCCAAATGAAGTGCAGGTCAAACCT
TGACAGTGACGACAAATCGTTGGGCGGGTCCAGGGCGAATTTTGCGACAACATGTCGAGGCTCAGCCGCTGT
CAGTCAACATGGTGGAGCACGACACTCTGGTCTACTCCAAAAATGTCAAAGATACAGTCTCAGAAGATCAAA
GGGCTATTGAGACTTTTCAACAAAGGATAATTTCGGGAAACCTCCTCGGATTCCATTGCCCAGCTATCTGTCAC
TTCATCGAAAGGACAGTAGAAAAGGAAGGTGGCTCCTACAAATGCCATCATTGCGATAAAGGAAAGGCTATC
ATTCAAGATCTCTCTGCCGACAGTGGTCCCAAAGATGGACCCCCACCCACGAGGAGCATCGTGGAAAAAGAA
GAGGTTCCAACCACGTCTACAAAGCAAGTGGATTGATGTGACATCTCCACTGACGTAAGGGATGACGCACAA
TCACACTATCCTTCGCAAGACCCTTCCTCTATATAAGGAAGTTCATTTCATTTGGAGAGGACACGCTCGAGTAT
AAGAGCTCATTTTTACAACAATTACCAACAACAACAAACAACAAACAACATTACAATTACATTTACAATTATCG
ATACAATGAAGTATATATAGACAATAAAAAAGTAGTATAATATATTATCAAATTATTATGAACAGTACATCTAT
GTCCTCATTGGGAGTGAGAAAAGGTTCATGGACTGATGAAGAAGATTTTCTTCTAAGAAAATGTATTGATAAG
TATGGTGAAGGAAAATGGCATCTTGTTCCCATAAGAGCTGGTAACTATTAAATTAACTATCACGTTATTTTTAT
TTGTCTTTCTGTCTCATTTTATTTGACGTTATTACGAATATCATCTGAAAATGTACGTGCAGGTCTGAATAGATG
TCGGAAAAGTTGTAGATTGAGGTGGCTGAATTATCTAAGGCCACATATCAAGAGAGGTGACTTTGAACAAGA
TGAAGTGGATCTCATTTTGAGGCTTCATAAGCTCTTAGGCAACAGGCATGCAAGTTTATGTTTTGACAAAATTT
GATTAGTATATATTATATATACGTGTGACTATTTCATCTAAATGTTACGTTATTTTACGTAGATGGTCACTTATT
GCTGGTAGACTTCCCGGAAGGACAGCTAACGATGTGAAAAACTATTGGAACACTAATCTTCTAAGGAAGTTA
AATACTACTAAAATTGTTCCTCGCGAAAAGATTAACAATAAGTGTGGAGAAATTAGTACTAAGATTGAAATTA
TAAAACCTCAACGACGCAAGTATTTCTCAAGCACAATGAAGAATGTTACAAACAATAATGTAATTTTGGACGA
GGAGGAACA

Blue font: 2x TALE binding site (scTALE ABL1 R); green font: upstream homologous arm; red font: pNOS-NptII-tOCS; orange font: CaMV 35S promoter; purple font: downstream homologous arm.

- ANT1D3 donor sequence

TTGGTCCCCAAGTACTTAAATTGGTCCCCAAGTACTTACCACAACACTTGTCGGTGAGATTATTTAATGCTGAT
TAGATTAGACAAAAATTAATTAGTTTTGAGTAGTGGCGTAAGTGTAAATAATTAGTCTCTTTTTTAACTTAGAA
AATAGTTTAATCCTTAGTATAAATAGTCAAAATCACTGGAATGAAAAACAGTTTTTAATTTTTCCAAATTTGATT
CTGATACCATGTTAAATTCGTGGTTCAAAATCACTGCAATGAAAAGAGCAATATTGTTTAACTTTTTTTAGGAA
AATCGAATTGATTTATAGTCAGTTGATATAGAGTGAATACATAAGGAACATATACAGTTGATACAATTGTATA
ATTCGTTCATACACTTAATACAAAGTGAACCCACAAGGAACATATACACTTAATATAATTGTATTCCTTGATAC
AAACCAATTTTGTTCGTGTCTCTACTCTCTATTTCAATTTCGCTTGACTCTTTACTTTTTCTAATATGTAGCTATAA
ATCGTAATTAAACAATACTATATCTCTAAATCTCTTATTAAGCTCAAACTATGGTCATATTCGAAAAAATCCTTT
TAAATATTGGTCCCCTCTCACGATTAATGATAGTTATAACTAACATTCAAATTTTAGTTGTACTTGACATCTAAA
ACTTAAAAAATAGTACAAGTTAACTTTTTCTTTTTTTTAAAAAAAGGAAATACTTGTATTTATTTTTTTAATATAT
AGTTATATTTTTGGTTATTTGAAAATACTTGATCTGTCATGTATGCTCAGTTAAATATCGTCACATTATAGAGAA
AAAAGTAATAGGAGAAAAAAATTAAAAATTATTTCGAAAAATCAAAATTTTTTTTTGATTGAAATGAAAGATG
GGTTTCCCAATCGAGGCTGGCAGGATAGGTACATTGGGAAATTTGGATTTGTGTGTTGAAAATGATTGTTCAA
TTTGGCTTTTATAACATTTGTCGTTTATAAGAATTCGGATCCGGAGCGGAGAATTAAGGGAGTCACGTTATGA

CCCCCGCCGATGACGCGGGACAAGCCGTTTTACGTTTGGAACTGACAGAACCGCAACGTTGAAGGAGCCACT
GAGCCGCGGGTTTCTGGAGTTTAATGAGCTAAGCACATACGTCAGAAACCATTATTGCGCGTTCAAAAGTCGC
CTAAGGTCACTATCAGCTAGCAAATATTTCTTGTCAAAAATGCTCCACTGACGTTCCATAAATTCCCCTCGGTA
TCCAATTAGAGTCTCATATTCACTCTCCTATTTTTACAACAATTACCAACAACAACAAACAACAAACAACATTAC
AATTACATTTACAATTACCATGGTTGAACAAGATGGATTGCACGCAGGTTCTCCGGCCGCTTGGGTGGAGAG
GCTATTCGGCTATGACTGGGCACAACAGACAATCGGCTGCTCTGATGCCGCCGTGTTCCGGCTGTCAGCGCA
GGGGCGCCCGGTTCTTTTTGTCAAGACCGACCTGTCCGGTGCCCTGAATGAACTGCAGGACGAGGCAGCGCG
GCTATCGTGGCTGGCCACGACGGGCGTTCCTTGCGCAGCTGTGCTCGACGTTGTCACTGAAGCGGGAAGGGA
CTGGCTGCTATTGGGCGAAGTGCCGGGGCAGGATCTCCTGTCATCTCACCTTGCTCCTGCCGAGAAAGTATCC
ATCATGGCTGATGCAATGCGGCGGCTGCATACGCTTGATCCGGCTACCTGCCCATTCGACCACCAAGCGAAAC
ATCGCATCGAGCGAGCACGTACTCGGATGGAAGCCGGTCTTGTCGATCAGGATGATCTGGACGAAGAGCATC
AGGGGCTCGCGCCAGCCGAACTGTTCGCCAGGCTCAAGGCGCGCATGCCCGACGGCGAGGATCTCGTCGTG
ACTCATGGCGATGCCTGCTTGCCGAATATCATGGTGGAAAATGGCCGCTTTTCTGGATTCATCGACTGTGGCC
GGCTGGGTGTGGCGGACCGCTATCAGGACATAGCGTTGGCTACCCGTGATATTGCTGAAGAGCTTGGCGGC
GAATGGGCTGACCGCTTCCTCGTGCTTTACGGTATCGCCGCTCCCGATTCGCAGCGCATCGCCTTCTATCGCCT
TCTTGACGAGTTCTTCTGAGCGGGACTCTGGGGTTCGCTAGAGTCCTGCTTTAATGAGATATGCGAGACGCCT
ATGATCGCATGATATTTGCTTTCAATTCTGTTGTGCACGTTGTAAAAAACCTGAGCATGTGTAGCTCAGATCCT
TACCGCCGGTTTCGGTTCATTCTAATGAATATATCACCCGTTACTATCGTATTTTTATGAATAATATTCTCCGTTC
AATTTACTGATTGTACCCTACTACTTATATGTACAATATTAAAATGAAAACAATATATTGTGCTGAATAGGTTTA
TAGCGACATCTATGATAGAGCGCCACAATAACAAACAATTGCGTTTTATTATTACAAATCCAATTTTAAAAAAA
GCGGCAGAACCGGTCAAACCTAAAAGACTGATTACATAAATCTTATTCAAATTTCAAAAGTGCCCCAGGGGCT
AGTATCTACGACACACCGAGCGGCGAACTAATAACGCTCACTGAAGGGAACTCCGGTTCCCCGCCGGCGCGC
ATGGGTGAGATTCCTTGAAGTTGAGTATTGGCCGTCCGCTCTACCGAAAGTTACGGGCACCATTCAACCCGGT
CCAGCACGGCGGCCGGGTAACCGACTTGCTGCCCCGAGAATTATGCAGCATTTTTTTGGTGTATGTGGGCCCC
AAATGAAGTGCAGGTCAAACCTTGACAGTGACGACAAATCGTTGGGCGGGTCCAGGGCGAATTTTGCGACAA
CATGTCGAGGCTCAGCCGCTGTCAGTCAACATGGTGGAGCACGACACTCTGGTCTACTCCAAAAATGTCAAAG
ATACAGTCTCAGAAGATCAAAGGGCTATTGAGACTTTTCAACAAAGGATAATTTCGGGAAACCTCCTCGGATT
CCATTGCCCAGCTATCTGTCACTTCATCGAAAGGACAGTAGAAAAGGAAGGTGGCTCCTACAAATGCCATCAT
TGCGATAAAGGAAAGGCTATCATTCAAGATCTCTCTGCCGACAGTGGTCCCAAAGATGGACCCCCACCCACGA
GGAGCATCGTGGAAAAAGAAGAGGTTCCAACCACGTCTACAAAGCAAGTGGATTGATGTGACATCTCCACTG
ACGTAAGGGATGACGCACAATCACACTATCCTTCGCAAGACCCTTCCTCTATATAAGGAAGTTCATTTCATTTG
GAGAGGACACGCTCGAGTATAAGAGCTCATTTTTACAACAATTACCAACAACAACAAACAACAAACAACATTA
CAATTACATTTACAATTATCGATACAATGAAGTATATATAGACAATAAAAAAGTAGTATAATATATTATCAAAT
TATTATGAACAGTACATCTATGTCCTCATTGGGAGTGAGAAAAGGTTCATGGACTGATGAAGAAGATTTTCTT
CTAAGAAAATGTATTGATAAGTATGGTGAAGGAAAATGGCATCTTGTTCCCATAAGAGCTGGTAACTATTAAA
TTAACTATCACGTTATTTTTATTTGTCTTTCTGTCTCATTTTATTTGACGTTATTACGAATATCATCTGAAAATGT
ACGTGCAGGTCTGAATAGATGTCGGAAAAGTTGTAGATTGAGGTGGCTGAATTATCTAAGGCCACATATCAA
GAGAGGTGACTTTGAACAAGATGAAGTGGATCTCATTTTGAGGCTTCATAAGCTCTTAGGCAACAGGCATGC
AAGTTTATGTTTTGACAAAATTTGATTAGTATATATTATATATACGTGTGACTATTTCATCTAAATGTTACGTTA
TTTTACGTAGATGGTCACTTATTGCTGGTAGACTTCCCGGAAGGACAGCTAACGATGTGAAAAACTATTGGAA
CACTAATCTTCTAAGGAAGTTAAATACTACTAAAATTGTTCCTCGCGAAAAGATTAACAATAAGTGTGGAGAA

ATTAGTACTAAGATTGAAATTATAAAACCTCAACGACGCAAGTATTTCTCAAGCACAATGAAGAATGTTACAA
ACAATAATGTAATTTTGGACGAGGAGGAACA

Blue font: 2x TALE binding site (scTALE ABL1 R); green font: upstream homologous arm; red font: pNOS-NptII-tOCS; orange font: CaMV 35S promoter; purple font: downstream homologous arm.

- ANT1D4 donor sequence

TTGGTCCCCAAGTACTTAAATTGGTCCCCAAGTACTTACCACAACACTTGTCGGTGAGATTATTTAATGCTGAT
TAGATTAGACAAAAATTAATTAGTTTTGAGTAGTGGCGTAAGTGTAAATAATTAGTCTCTTTTTTAACTTAGAA
AATAGTTTAATCCTTAGTATAAATAGTCAAAATCACTGGAATGAAAAACAGTTTTTAATTTTTCCAAATTTGATT
CTGATACCATGTTAAATTCGTGGTTCAAAATCACTGCAATGAAAAGAGCAATATTGTTTAACTTTTTTTAGGAA
AATCGAATTGATTTATAGTCAGTTGATATAGAGTGAATACATAAGGAACATATACAGTTGATACAATTGTATA
ATTCGTTCATACACTTAATACAAAGTGAACCCACAAGGAACATATACACTTAATATAATTGTATTCCTTGATAC
AAACCAATTTTGTTCGTGTCTCTACTCTCTATTTCAATTTCGCTTGACTCTTTACTTTTTCTAATATGTAGCTATAA
ATCGTAATTAAACAATACTATATCTCTAAATCTCTTATTAAGCTCAAACTATGGTCATATTCGAAAAAATCCTTT
TAAATATTGGTCCCCTCTCACGATTAATGATAGTTATAACTAACATTCAAATTTTAGTTGTACTTGACATCTAAA
ACTTAAAAAATAGTACAAGTTAACTTTTTCTTTTTTTTAAAAAAAGGAAATACTTGTATTTATTTTTTTAATATAT
AGTTATATTTTTGGTTATTTGAAAATACTTGATCTGTCATGTATGCTCAGTTAAATATCGTCACATTATAGAGAA
AAAAGTAATAGGAGAAAAAAATTAAAAATTATTTCGAAAAATCAAAATTTTTTTTTGATTGAAATGAAAGATG
GGTTTCGAATTCGGATCCGGAGCGGAGAATTAAGGGAGTCACGTTATGACCCCCGCCGATGACGCGGGACA
AGCCGTTTTACGTTTGGAACTGACAGAACCGCAACGTTGAAGGAGCCACTGAGCCGCGGGTTTCTGGAGTTT
AATGAGCTAAGCACATACGTCAGAAACCATTATTGCGCGTTCAAAAGTCGCCTAAGGTCACTATCAGCTAGCA
AATATTTCTTGTCAAAAATGCTCCACTGACGTTCCATAAATTCCCCTCGGTATCCAATTAGAGTCTCATATTCAC
TCTCCTATTTTTACAACAATTACCAACAACAACAAACAACAAACAACATTACAATTACATTTACAATTACCATGG
TTGAACAAGATGGATTGCACGCAGGTTCTCCGGCCGCTTGGGTGGAGAGGCTATTCGGCTATGACTGGGCAC
AACAGACAATCGGCTGCTCTGATGCCGCCGTGTTCCGGCTGTCAGCGCAGGGGCGCCCGGTTCTTTTTGTCAA
GACCGACCTGTCCGGTGCCCTGAATGAACTGCAGGACGAGGCAGCGCGGCTATCGTGGCTGGCCACGACGG
GCGTTCCTTGCGCAGCTGTGCTCGACGTTGTCACTGAAGCGGGAAGGGACTGGCTGCTATTGGGCGAAGTGC
CGGGGCAGGATCTCCTGTCATCTCACCTTGCTCCTGCCGAGAAAGTATCCATCATGGCTGATGCAATGCGGCG
GCTGCATACGCTTGATCCGGCTACCTGCCCATTCGACCACCAAGCGAAACATCGCATCGAGCGAGCACGTACT
CGGATGGAAGCCGGTCTTGTCGATCAGGATGATCTGGACGAAGAGCATCAGGGGCTCGCGCCAGCCGAACT
GTTCGCCAGGCTCAAGGCGCGCATGCCCGACGGCGAGGATCTCGTCGTGACTCATGGCGATGCCTGCTTGCC
GAATATCATGGTGGAAAATGGCCGCTTTTCTGGATTCATCGACTGTGGCCGGCTGGGTGTGGCGGACCGCTA
TCAGGACATAGCGTTGGCTACCCGTGATATTGCTGAAGAGCTTGGCGGCGAATGGGCTGACCGCTTCCTCGT
GCTTTACGGTATCGCCGCTCCCGATTCGCAGCGCATCGCCTTCTATCGCCTTCTTGACGAGTTCTTCTGAGCGG
GACTCTGGGGTTCGCTAGAGTCCTGCTTTAATGAGATATGCGAGACGCCTATGATCGCATGATATTTGCTTTC
AATTCTGTTGTGCACGTTGTAAAAAACCTGAGCATGTGTAGCTCAGATCCTTACCGCCGGTTTCGGTTCATTCT
AATGAATATATCACCCGTTACTATCGTATTTTTATGAATAATATTCTCCGTTCAATTTACTGATTGTACCCTACTA
CTTATATGTACAATATTAAAATGAAAACAATATATTGTGCTGAATAGGTTTATAGCGACATCTATGATAGAGC
GCCACAATAACAAACAATTGCGTTTTATTATTACAAATCCAATTTTAAAAAAAGCGGCAGAACCGGTCAAACC
TAAAAGACTGATTACATAAATCTTATTCAAATTTCAAAAGTGCCCCAGGGGCTAGTATCTACGACACACCGAG
CGGCGAACTAATAACGCTCACTGAAGGGAACTCCGGTTCCCCGCCGGCGCGCATGGGTGAGATTCCTTGAAG
TTGAGTATTGGCCGTCCGCTCTACCGAAAGTTACGGGCACCATTCAACCCGGTCCAGCACGGCGGCCGGGTA

ACCGACTTGCTGCCCCGAGAATTATGCAGCATTTTTTTGGTGTATGTGGGCCCCAAATGAAGTGCAGGTCAAA
CCTTGACAGTGACGACAAATCGTTGGGCGGGTCCAGGGCGAATTTTGCGACAACATGTCGAGGCTCAGCCGC
TGTCAGTCAACATGGTGGAGCACGACACTCTGGTCTACTCCAAAAATGTCAAAGATACAGTCTCAGAAGATCA
AAGGGCTATTGAGACTTTTCAACAAAGGATAATTTCGGGAAACCTCCTCGGATTCCATTGCCCAGCTATCTGTC
ACTTCATCGAAAGGACAGTAGAAAAGGAAGGTGGCTCCTACAAATGCCATCATTGCGATAAAGGAAAGGCTA
TCATTCAAGATCTCTCTGCCGACAGTGGTCCCAAAGATGGACCCCCACCCACGAGGAGCATCGTGGAAAAAG
AAGAGGTTCCAACCACGTCTACAAAGCAAGTGGATTGATGTGACATCTCCACTGACGTAAGGGATGACGCAC
AATCACACTATCCTTCGCAAGACCCTTCCTCTATATAAGGAAGTTCATTTCATTTGGAGAGGACACGCTCGAGT
ATAAGAGCTCATTTTTACAACAATTACCAACAACAACAAACAACAAACAACATTACAATTACATTTACAATTAT
CGATACAATGAAGTATATATAGACAATAAAAAAGTAGTATAATATATTATCAAATTATTATGAACAGTACATCT
ATGTCCTCATTGGGAGTGAGAAAAGGTTCATGGACTGATGAAGAAGATTTTCTTCTAAGAAAATGTATTGATA
AGTATGGTGAAGGAAAATGGCATCTTGTTCCCATAAGAGCTGGTAACTATTAAATTAACTATCACGTTATTTTT
ATTTGTCTTTCTGTCTCATTTTATTTGACGTTATTACGAATATCATCTGAAAATGTACGTGCAGGTCTGAATAGA
TGTCGGAAAAGTTGTAGATTGAGGTGGCTGAATTATCTAAGGCCACATATCAAGAGAGGTGACTTTGAACAA
GATGAAGTGGATCTCATTTTGAGGCTTCATAAGCTCTTAGGCAACAGGCATGCAAGTTTATGTTTTGACAAAA
TTTGATTAGTATATATTATATATACGTGTGACTATTTCATCTAAATGTTACGTTATTTTACGTAGATGGTCACTT
ATTGCTGGTAGACTTCCCGGAAGGACAGCTAACGATGTGAAAAACTATTGGAACACTAATCTTCTAAGGAAGT
TAAATACTACTAAAATTGTTCCTCGCGAAAAGATTAACAATAAGTGTGGAGAAATTAGTACTAAGATTGAAAT
TATAAAACCTCAACGACGCAAGTATTTCTCAAGCACAATGAAGAATGTTACAAACAATAATGTAATTTTGGAC
GAGGAGGAACA

Blue font: 2x TALE binding site (scTALE ABL1 R); green font: upstream homologous arm; red font: pNOS-NptII-tOCS; orange font: CaMV 35S promoter; purple font: downstream homologous arm.

- **crRNA expression cassettes and donor sequences for SlHKT1;2 locus**
  - U6-crR1.20$^{HKT1;2}$ expression cassette:

TGATCAAAAGTCCCACATCGATCAGGTGATATATAGCAGCTTAGTTTATATAATGATAGAGTCGACATAGCGA
TTGtAATTTCTACTAAGTGTAGATACTATTCACCACAGTATCAATTTTTTT

Red font: AtU6 promoter; green font: LbCas12a scaffold; light blue font: LbCas12a_gRNA1, 20nt; black G: transcription start; black TTTTTT: termination sequence.

  - U6-crR1.23$^{HKT1;2}$ expression cassette:

TGATCAAAAGTCCCACATCGATCAGGTGATATATAGCAGCTTAGTTTATATAATGATAGAGTCGACATAGCGA
TTGtAATTTCTACTAAGTGTAGATACTATTCACCACAGTATCAACTTTTTTTT

Red font: AtU6 promoter; green font: LbCas12a scaffold; blue font: LbCas12a_gRNA1, 23nt; black G: transcription start; black TTTTTT: termination sequence.

  - U6-crR2.20$^{HKT1;2}$ expression cassette:

TGATCAAAAGTCCCACATCGATCAGGTGATATATAGCAGCTTAGTTTATATAATGATAGAGTCGACATAGCGA
TTGtAATTTCTACTAAGTGTAGATCCTACAAATGAAAACATGATTTTTTTT

Red font: AtU6 promoter; green font: LbCas12a scaffold; orange font: LbCas12a_gRNA2, 20nt; black G: transcription start; black TTTTTT: termination sequence.

  - U6-crR2.23$^{HKT1;2}$ expression cassette:

TGATCAAAAGTCCCACATCGATCAGGTGATATATAGCAGCTTAGTTTATATAATGATAGAGTCGACATAGCGA TTGtAATTTCTACTAAGTGTAGATCCTACAAATGAAAACATGATGATTTTTTTT

Red font: AtU6 promoter; green font: LbCas12a scaffold; purple font: LbCas12a_gRNA2, 23nt; black G: transcription start; black TTTTTT: termination sequence.

- U6-crR1-2.20$^{HKT1;2}$ expression cassette:

TGATCAAAAGTCCCACATCGATCAGGTGATATATAGCAGCTTAGTTTATATAATGATAGAGTCGACATAGCGA TTGtAATTTCTACTAAGTGTAGATACTATTCACCACAGTATCAATAATTTCTACTAAGTGTAGATCCTACAAATG AAAACATGATTTTTTT

Red font: AtU6 promoter; green font: LbCas12a scaffolds; light blue font: LbCas12a_gRNA1, 20nt; orange font: LbCas12a_gRNA2, 20nt; black G: transcription start; black TTTTTT: termination sequence.

- U6-crR1-2.23$^{HKT1;2}$ expression cassette:

TGATCAAAAGTCCCACATCGATCAGGTGATATATAGCAGCTTAGTTTATATAATGATAGAGTCGACATAGCGA TTGtAATTTCTACTAAGTGTAGATACTATTCACCACAGTATCAACTTTAATTTCTACTAAGTGTAGATCCTACAA ATGAAAACATGATGATTTTTTT

Red font: AtU6 promoter; green font: LbCas12a scaffolds; blue font: LbCas12a_gRNA1, 23nt; purple font: LbCas12a_gRNA2, 23nt; black G: transcription start; black TTTTTT: termination sequence.

- HKT1;2 donor (HKTD1) sequence

GGAGACTCATGAACTTGAAATGCATAGAATGCCTATTTTAACACGTTTTTAACAAATAATAAATAAGTTGTATA GCATCTTATAATTCCACTAACTCTATGATAAGGAAATATCAGTGATCATTTGGCAAGAATTATTATATTCTAACT CCTTTTGGTACGTACCATATGTTGTTGGGATTAATTTAAATCATTGGTAAGAATTATTATATTCTAACTCCTTTT GATACGTACCATATGTTATTGGGATTAATTTAAAGCTTACACAATGTGTCTAGTATAGGCATTCCACTACACAA TTTTTACTATAAATTGAACCCTTGAGTATTATAATAAAACTTAAGAGACAAACAAATTTATTTTACTAATGAAGT CATCACTTTCAATTTCTTTCTTTAAGATTATGATGAGTCTTAGGGTAAAGCCATTTTGGATTGAATTAGGGTATT TCACAACCCTTTCATTGTTAGGTTTCTTAGCTTTGAATTATGTTTCAAAACCTAGAACCCTACCGTCTTTTCGTCC TCAAAACCTAGATGTTCTTTTCACTTCTGTTTCTTCAACCACAGTTTCTAGTATGTCCACTATTGAAATGGAAGT TTTCTCAAATGTTCAACTTGTTTTCATGACCATATTGATGTTTCTTGGTGGGGAAGCTTTTACCTCTTTTCTTAGC CTTAAACTCATCAAGAATAAAGAAAGCAAAGATAAATCTTTTAGTAACAAAGATTATGAGCTAGGGAATGTAA TAAACGTTGACAATAAGTTAGAAGATGTGATAATAATAAACCCTATCGAAGATCATATTCATGATCATCACGA TGAAATTATAAAGATTAAATCGATAAAATTGTTGAGTAATGTGGTTTTTGGATATATTCTTGTTGTTATTCTTCT TGGTTCATCGTTAGTTTCTCTCTATATAATAATCATCCCTAGCGCCAAACAAATCCTTGACCAAAAAGGCCTTAA TTTACATACTTTCTCTCTATTCACCACAGTATCAACTTTTGCAGATTGTGGTTTCTTACCAACAAATGAAAACAT GATGATTTTCAAGAAAAATTCAGGACTTCTTCTCATTCTTATCCCTCAAGTCCTTCTAGGGAACACTTTGTTTGC TCCTTGTTTACGCATCGTTATAATGTTCTTATGGAAAATCACAAAGAGACATGAGTATGAGTATATTTTGAAGA ACTCAAAATGTGTTGGATATTCACATATTTTTCCAAGTTATGAAACAATTGGTATTGCTATTACTGTTGTGGGA TTAATAGTATTTCAATTTGTTATGTTTTGTTCATTGGAGTGGAATTCTGAAGGTACTTCTGGATTAAGTACTTAT GAGAAGATTGTGGGATCTTTGTTTGAAGTTGTGAATACAAGGCATGCTGGTCTATCTGTATTTGATCTTTCAAC TTTTACTCCATCAATCTTAGTATTGTTTGCCTTAATGATGTAAGTATTCTCTACAATCTATGTTCTTTCGTGTTCT GTCTCAATTTACGTGACATATATACTACTTCTTTGTTTTATATCTGTGTCTCTGGAAGAATTAATGTCAACTTTCT ATAGTTAGAAATAACTTAATTTTCAAATTCCCTTTTTATCTTTGATTCACATGAATATCTAAGATTTGCTTTATAT TGCAAATTTTCAAATTTGATGTTAAGTTATTAAAAAGTATCACATGAACAAGAGAGAGGAGAAAGTAGTTCAT

TGTTTAGAAATTTATCATCCACATGCAACTATGTTAATACTAACATATTTGTTTAAGTTGTTGTAATAGGATATG
ATAAACTTTTAACAACAAATTAAGTTTAACTATTAAACAAATGTGTTAAAAGTGTTA

Purple font: LbCas12a_gHKT12.1; orange font: LbCas12a_gHKT12.2; yellow highlighted font: modified PAM and LbCas12a gRNA core region to prevent recut after HDR editing; red font: N217D (AAT to GAT).

- **crRNA expression cassettes and donor sequences for SlEPSPS1 locus**
  - Dual U6-crR1-2.23 EPSPS1 expression cassette:

TGATCAAAAGTCCCACATCGATCAGGTGATATATAGCAGCTTAGTTTATATAATGATAGAGTCGACATAGCGA
TTGtAATTTCTACTAAGTGTAGATCAAGGAATAGTTGGATTTCTTCCTAATTTCTACTAAGTGTAGATAATCGTT
CCTTCTTCGTGCCATTTTTTTT

Red font: AtU6 promoter; green font: LbCas12a scaffolds; blue font: LbCas12a_gRNA1, 23nt; purple font: LbCas12a_gRNA2, 23nt; black G: transcription start; black TTTTTT: termination sequence.

  - Dual U6-crR1-3.23 EPSPS1 expression cassette:

TGATCAAAAGTCCCACATCGATCAGGTGATATATAGCAGCTTAGTTTATATAATGATAGAGTCGACATAGCGA
TTGtAATTTCTACTAAGTGTAGATCAAGGAATAGTTGGATTTCTTCCTAATTTCTACTAAGTGTAGATAATCTCC
ACATCTCCTAGAGCCATTTTTTT

Red font: AtU6 promoter; green font: LbCas12a scaffolds; blue font: LbCas12a_gRNA1, 23nt; orange font: LbCas12a_gRNA3, 23nt; black G: transcription start; black TTTTTT: termination sequence.

  - Dual U6-gR2-4.23 EPSPS1 expression cassette:

TGATCAAAAGTCCCACATCGATCAGGTGATATATAGCAGCTTAGTTTATATAATGATAGAGTCGACATAGCGA
TTGtAATTTCTACTAAGTGTAGATAATCGTTCCTTCTTCGTGCCATTTAATTTCTACTAAGTGTAGATCTCCAAAA
GTCTCTTGTCCCTCCTTTTTT

Red font: AtU6 promoter; green font: LbCas12a scaffolds; purple font: LbCas12a_gRNA2, 23nt; light blue font: LbCas12a_gRNA4, 23nt; black G: transcription start; black TTTTTT: termination sequence.

  - SlEPSPS1 donor 1-1 (EPSPS1D1) sequence

TTGGTCCCCAAGTACTTAAATTGGTCCCCAAGTACTTTTCCTGAGCTTAAGGTAATCAAACACTAATAAACATT
TTCTTTGAGATCTGTCGTACCAAACATACTGAGTTTATATATTTGGTTCCAATGGTTTTGATACTTCATTTAAAT
ACCATGTCACCTGCAATGAGCTTAAATATGCTTTTTACCACTTCAAGAAACATTGGTATTTAGCTGATTTTTGAA
GTGCTACTTGCAATACTAATATATTAATATGTTAAAAGACATAAATTGTTTGCACATCTTATTTTTTGCTTTATG
TTTTGTTTTTATTGAAATGATTGCATTTTGTCAATCTGTCTACCAAATTGATCTGCCAAATGTTCAACCTTTGTGA
CATTTTGTTGTGGTTCTGGGGATTCAGCAATTCCCTTTTTGGAGTTTCTGACCCTACCAGGTTGGCATTAGGGT
GGGTTACTTATAAATTTGGACAATGGTTTTTTTTCTTAATTAATTCAAGGAGATATTTGTTAGAGTACCATCATT
AATGATTTATGCAAATCAACGCAAATTAAGTATATGGTAAAAACTGATGCTATAAGTGTGAGGAGAGGAGCT
AAGGGTTGAAAGAAACACTTAATTCTTAGTATAAGCTGTCCTAAACGACTGGGAACTCTCTTCCCAAGCTCAT
GGTGTCCTGAACAACTCATACAGTATAATACACTCATAGCCATCGAGTGTGCAACCTTTTTAAGCTGACAACTA
TATTCACGTTCCATTGTTCTTTAACTTATCGTGTACATTTTCATGCTTTCATAATCTTGATATGAGTTACTTACAA
CCTTGTGCTGCCATTACATGTCACTGGCACTTGAATTTCAGGGAAGGACTGTTGTTGACAATTTACTGAGTAGT
GACGACATTCATTACATGCTTGGTGCGTTGAAAACACTTGGACTTCATGTTGAAGATGACAATGAAAACCAAC

GAGCAATTGTGGAAGGTTGTGGTGGGCAGTTTCCTGTCGGTAAAAAGTCTGAGGAAGAAATCCAACTATTCT
TAGGTAATGCAGCTACAGCAATGCGTCCGTTGACAGCAGCAGTTACTGTAGCTGGAGGACATTCAAGGTCTTT
CAATCGTTCCTTCTTCGTGCCATTTGTTTTTGTCTTGTTTGCTTTTTTATTTTTTCTTCTTTTTTTCCAGTGTTGTTT
CTTTGTCTTTTTTTTTTTTTTGGGGGGGGGGGGTGCTGTTTCTTTTGAAGCCATTTTGATAGTCTCATGAATTT
GTATTTTTGGGAATGGTGGAAAACTTCTGAAATGTACATCGATAATTTAATTTTTTTTTGTTAGATAATAGTTG
CCCTGGTGATAAATTACATGTCAGTTAGCTCCTCAAAGGTTCAGTAATCTGGATGTGGTGCTTTGAGAGCTTC
AGAAATGCATGCATACTTTGTACGTCATGTGTTTTACCATCTTTCTCAACGTGACAGAACAAGACAGGAAACG
AGCATATTTTTGTTTGCTTCTTCTTTAGTTAGAGTTGTTTTTCTTTTCTTTTTTTTGGTCTAGAAAATATTACCTTG
TGGTGGGTGATAACTCTATGAATAATTGTGTGCTACAGATATGTTCTTGATGGAGTTCCTAGGATGAGAGAAA
GACCAATTGGTGATTTGGTTGATGGTCTTAAGCAGCTTGGCGCAGAGGTAGATTGTTCCCTTGGTACGAATTG
TCCCCCAGTTCGAATTGTCAGCAAGGGAGGACTTCCAGGAGGGAAGGTGAGGATTATGATTTTATTACGTAT
AATGTTAGTGATTGGCAGCCTGTTACTGCAATTTTCTTAACTAGGAATAGTATTATCACTATTATTTTAAAAAA
AGAAAAAAGGCATAGTATTGTCACTATGATGAGTAATTTTTTAAATGCAATAGCATTTTTTCTTGAAAAGTTAA
ACATACCTTAAACTTCGGAGAAACTGGCTTACATTATCTTATTTTAGATCCTGAGTAATAAAGCCAAAGATCCC
TCTCATCAGTTGGATACGATGAGTTATAGATGGAAATGAGTGGTGGTGAGACTTGAACCTGAAACCTCAACCT
TGTTCTGGTACCATCTTGAATTATGTGTACAGCCTCTCTATAAAGCTTCAGCTATAAGAGAGGGCACACCTATA
TTTCATTTGATTGTATTGTAACAAACATATTTTTTAATCTCCATCTGATTTACCTTCATTTTATTTCATTTTATACT
TTTTCTTAGAACTACTGACCTTTAAGTCTAAGAACAAGTATGCACTCACTCCCTCGTGCCATAGGTTAAATTAG
TTTATAGTCAATATGTTTTCTTGCATGTATCAAGCTCGTATGATAACAAGCTATTCAACTCTAGTATCTATCCAC
TTAACGAGAAGTCCTAAATGGGAAAGGCAGACTTCCAATTTTTCAAGTAACTTGCTGTCCTCCTTTATTTTGTC
TTTGTTTCCTCCAAAAGTCTCTTGTCCCTCCATTTGATCTCTTCCACATTCCTTGTCGCTGCCACTAAATTAAAGA
AGATGCATTTGATTCTGCAATATTATTTTATATTGATGAATCATGTTCATATGTTGTCTGTGGTTTACTTGATCA
GCTTCTTTCCTAGCATGGAAAGATGTAATATAACCGTTTTTGAGTTCTTGCTAAGTTATACTCCCTTCATATCTA
TCAGGTAAAGCTCTCTGGATCCATCAGCAGCCAATACCTGACTGCTCTGCTTATGGCTGCTCCCCTGACTCTAG
GAGATGTGGAGATCGAGATAATTGACAAACTGATATCTGTGCCTTATGTTGAAATGACACTGAAGTTGATGG
AGCGATTTGGTGTCTTTGTGGAGCACAGTAGTGGCTGGGACAGATTCTTGGTAAAAGGAGGTCAGAAGTACA
AGTAAGTCGGCTTTCACACTGTTTTAATGTCTTTAATAATCTGGTTCCACCGATCAATGAAGCTGACAGTTACG
AAAATATCATAGATCTCCTGGGAAAGCATTTGTTGAAGGAGATGCCTCAAGTGCTAGCTATTTTTTGGCGGGG
GCAGCAGTCACAGGTGGAACCGTCACTGTTGAAGGTTGTGGAACAAGCAGTTTACAGGTATTTCTCATGTCAT
TAAGACCTTGGGGGCAATCACATTCTAGACTTTTCCATCTTGTGCTTGTATTTGTGACAGCGCCATAATACGTT
CTATTTCTCTTCTGTTCACTAGCTTAATTTGTGGAAGTACTAAAAAGTAAGAATTGCACAGTGCATGGCCTACA
TCTTAGTGTATAACTGCATAAGGTAATTTTCTAACAAGGGACTTACTTTGAGCTTTATGTTTCCTTTGTGAAGG
GAGATGTTAAGTTCGCTGAGGTCCTCGAGAAGATGGGGGCAGAAGTTACATGGACAGAGAACAGTGTCACA
GTTAAAGGACCTCCGAGGAACTCTTCTGGAATGAAACATTTGCGTGCCATTGACGTGAACATGAACAAAATGC
CAGATGTGGCCATGACTCTTGCCGTAGTTGCACTTTTTGCTGATGGTCCTACTACCATAAGAGATGGTATGGCT
AACCTTGTTTCCCTGGTATCAAATCCTATTGAAGCACTTCGTTTAGATTAAGATATTATTCTGAAGCGATTGGG
ACCTGGGGCTCTATTCTCCAATATGAAAGCATTTCCCTGCATAATTAGTTTTTTTAAATGCTGTGATAACCTGAC
TAAAATGTGCAAAGAGGTTTTCCTGGTAGTTTAGGGGTCTG

Blue font: 2x TALE binding site (scTALE ABL1 R); yellow highlighted font: G177A; turquoise highlighted font: A268T; red fonts: base changes (without changing a.a coding) for modification of

gRNA binding sequences and PAM; orange fonts: red fonts: base changes (without changing a.a coding) for modification of BpiI, BsaI or BsmBI restriction sites for facilitating Golden gate cloning.

- SlEPSPS1 donor 1-3 (EPSPS1D3) sequence

TTGGTCCCCAAGTACTTAAATTGGTCCCCAAGTACTTTTCCTGAGCTTAAGGTAATCAAACACTAATAAACATT
TTCTTTGAGATCTGTCGTACCAAACATACTGAGTTTATATATTTGGTTCCAATGGTTTTGATACTTCATTTAAAT
ACCATGTCACCTGCAATGAGCTTAAATATGCTTTTTACCACTTCAAGAAACATTGGTATTTAGCTGATTTTTGAA
GTGCTACTTGCAATACTAATATATTAATATGTTAAAAGACATAAATTGTTTGCACATCTTATTTTTTGCTTTATG
TTTTGTTTTTATTGAAATGATTGCATTTTGTCAATCTGTCTACCAAATTGATCTGCCAAATGTTCAACCTTTGTGA
CATTTTGTTGTGGTTCTGGGGATTCAGCAATTCCCTTTTTGGAGTTTCTGACCCTACCAGGTTGGCATTAGGGT
GGGTTACTTATAAATTTGGACAATGGTTTTTTTTCTTAATTAATTCAAGGAGATATTTGTTAGAGTACCATCATT
AATGATTTATGCAAATCAACGCAAATTAAGTATATGGTAAAAACTGATGCTATAAGTGTGAGGAGAGGAGCT
AAGGGTTGAAAGAAACACTTAATTCTTAGTATAAGCTGTCCTAAACGACTGGGAACTCTCTTCCCAAGCTCAT
GGTGTCCTGAACAACTCATACAGTATAATACACTCATAGCCATCGAGTGTGCAACCTTTTTAAGCTGACAACTA
TATTCACGTTCCATTGTTCTTTAACTTATCGTGTACATTTTCATGCTTTCATAATCTTGATATGAGTTACTTACAA
CCTTGTGCTGCCATTACATGTCACTGGCACTTGAATTTCAGGGAAGGACTGTTGTTGACAATTTACTGAGTAGT
GACGACATTCATTACATGCTTGGTGCGTTGAAAACACTTGGACTTCATGTTGAAGATGACAATGAAAACCAAC
GAGCAATTGTGGAAGGTTGTGGTGGGCAGTTTCCTGTCGGTAAAAAGTCTGAGGAAGAAATCCAACTATTCT
TAGGTAATGCAGGAATCGCAATGCGTTCTTTGACAGCAGCAGTTACTGTAGCTGGAGGACATTCAAGGTCTAT
CCATCGTTCCTTCTTCGTGCCATTTGTTTTTGTCTTGTTTGCTTTTTTATTTTTTCTTCTTTTTTTCCAGTGTTGTTT
CTTTGTCTTTTTTTTTTTTTTTGGGGGGGGGGGGGTGCTGTTTCTTTTGAAGCCATTTTGATAGTCTCATGAATTT
GTATTTTTGGGAATGGTGGAAAACTTCTGAAATGTACATCGATAATTTAATTTTTTTTTGTTAGATAATAGTTG
CCCTGGTGATAAATTACATGTCAGTTAGCTCCTCAAAGGTTCAGTAATCTGGATGTGGTGCTTTGAGAGCTTC
AGAAATGCATGCATACTTTGTACGTCATGTGTTTTACCATCTTTCTCAACGTGACAGAACAAGACAGGAAACG
AGCATATTTTTGTTTGCTTCTTCTTTAGTTAGAGTTGTTTTTCTTTTCTTTTTTTTGGTCTAGAAAATATTACCTTG
TGGTGGGTGATAACTCTATGAATAATTGTGTGCTACAGATATGTTCTTGATGGAGTTCCTAGGATGAGAGAAA
GACCAATTGGTGATTTGGTTGATGGTCTTAAGCAGCTTGGCGCAGAGGTAGATTGTTCCCTTGGTACGAATTG
TCCCCCAGTTCGAATTGTCAGCAAGGGAGGACTTCCAGGAGGGAAGGTGAGGATTATGATTTTATTACGTAT
AATGTTAGTGATTGGCAGCCTGTTACTGCAATTTTCTTAACTAGGAATAGTATTATCACTATTATTTTAAAAAA
AGAAAAAAGGCATAGTATTGTCACTATGATGAGTAATTTTTTAAATGCAATAGCATTTTTTCTTGAAAAGTTAA
ACATACCTTAAACTTCGGAGAAACTGGCTTACATTATCTTATTTTAGATCCTGAGTAATAAAGCCAAAGATCCC
TCTCATCAGTTGGATACGATGAGTTATAGATGGAAATGAGTGGTGGTGAGACTTGAACCTGAAACCTCAACCT
TGTTCTGGTACCATCTTGAATTATGTGTACAGCCTCTCTATAAAGCTTCAGCTATAAG

Blue font: 2x TALE binding site (scTALE ABL1 R); yellow highlighted font: T178I; turquoise highlighted font: P182S; red fonts: base changes (without changing a.a coding) for modification of gRNA binding sequences and PAM; orange fonts: red fonts: base changes (without changing a.a coding) for modification of BpiI, BsaI or BsmBI restriction sites for facilitating Golden gate cloning.

- SlEPSPS1 donor 1-4 (EPSPS1D4) sequence

TTGGTCCCCAAGTACTTAAATTGGTCCCCAAGTACTTTTCCTGAGCTTAAGGTAATCAAACACTAATAAACATT
TTCTTTGAGATCTGTCGTACCAAACATACTGAGTTTATATATTTGGTTCCAATGGTTTTGATACTTCATTTAAAT
ACCATGTCACCTGCAATGAGCTTAAATATGCTTTTTACCACTTCAAGAAACATTGGTATTTAGCTGATTTTTGAA
GTGCTACTTGCAATACTAATATATTAATATGTTAAAAGACATAAATTGTTTGCACATCTTATTTTTTGCTTTATG
TTTTGTTTTTATTGAAATGATTGCATTTTGTCAATCTGTCTACCAAATTGATCTGCCAAATGTTCAACCTTTGTGA
CATTTTGTTGTGGTTCTGGGGATTCAGCAATTCCCTTTTTGGAGTTTCTGACCCTACCAGGTTGGCATTAGGGT
GGGTTACTTATAAATTTGGACAATGGTTTTTTTCTTAATTAATTCAAGGAGATATTTGTTAGAGTACCATCATT
AATGATTTATGCAAATCAACGCAAATTAAGTATATGGTAAAAACTGATGCTATAAGTGTGAGGAGAGGAGCT
AAGGGTTGAAAGAAACACTTAATTCTTAGTATAAGCTGTCCTAAACGACTGGGAACTCTCTTCCCAAGCTCAT
GGTGTCCTGAACAACTCATACAGTATAATACACTCATAGCCATCGAGTGTGCAACCTTTTTAAGCTGACAACTA
TATTCACGTTCCATTGTTCTTTAACTTATCGTGTACATTTTCATGCTTTCATAATCTTGATATGAGTTACTTACAA
CCTTGTGCTGCCATTACATGTCACTGGCACTTGAATTTCAGGGAAGGACTGTTGTTGACAATTTACTGAGTAGT
GACGACATTCATTACATGCTTGGTGCGTTGAAAACACTTGGACTTCATGTTGAAGATGACAATGAAAACCAAC
GAGCAATTGTGGAAGGTTGTGGTGGGCAGTTTCCTGTCGGTAAAAAGTCTGAGGAAGAAATCCAACTATTCT
TAGGTAATGCAGCTACAGCAATGCGTCCGTTGACAGCAGCAGTTACTGTAGCTGGAGGACATTCAAGGTCTG
TCCATCGTTCCTTCTTCGTGCCATTTGTTTTTGTCTTGTTTGCTTTTTTATTTTTTCTTCTTTTTTTCCAGTGTTGTT
TCTTTGTCTTTTTTTTTTTTTTGGGGGGGGGGGGGTGCTGTTTCTTTTGAAGCCATTTTGATAGTCTCATGAATT
TGTATTTTTGGGAATGGTGGAAAACTTCTGAAATGTACATCGATAATTTAATTTTTTTTTGTTAGATAATAGTTG
CCCTGGTGATAAATTACATGTCAGTTAGCTCCTCAAAGGTTCAGTAATCTGGATGTGGTGCTTTGAGAGCTTC
AGAAATGCATGCATACTTTGTACGTCATGTGTTTTACCATCTTTCTCAACGTGACAGAACAAGACAGGAAACG
AGCATATTTTTGTTTGCTTCTTCTTTAGTTAGAGTTGTTTTTCTTTTCTTTTTTTTGGTCTAGAAAATATTACCTTG
TGGTGGGTGATAACTCTATGAATAATTGTGTGCTACAGATATGTTCTTGATGGAGTTCCTAGGATGAGAGAAA
GACCAATTGGTGATTTGGTTGATGGTCTTAAGCAGCTTGGCGCAGAGGTAGATTGTTCCCTTGGTACGAATTG
TCCCCCAGTTCGAATTGTCAGCAAGGGAGGACTTCCAGGAGGGAAGGTGAGGATTATGATTTTATTACGTAT
AATGTTAGTGATTGGCAGCCTGTTACTGCAATTTTCTTAACTAGGAATAGTATTATCACTATTATTTTAAAAAA
AGAAAAAAGGCATAGTATTGTCACTATGATGAGTAATTTTTTAAATGCAATAGCATTTTTTCTTGAAAAGTTAA
ACATACCTTAAACTTCGGAGAAACTGGCTTACATTATCTTATTTTAGATCCTGAGTAATAAAGCCAAAGATCCC
TCTCATCAGTTGGATACGATGAGTTATAGATGGAAATGAGTGGTGGTGAGACTTGAACCTGAAACCTCAACCT
TGTTCTGGTACCATCTTGAATTATGTGTACAGCCTCTCTATAAAGCTTCAGCTATAAGAGAGGGCACACCTATA
TTTCATTTGATTGTATTGTAACAAACATATTTTTTAATCTCCATCTGATTTACCTTCATTTTATTTCATTTTATACT
TTTTCTTAGAACTACTGACCTTTAAGTCTAAGAACAAGTATGCACTCACTCCCTCGTGCCATAGGTTAAATTAG
TTTATAGTCAATATGTTTTCTTGCATGTATCAAGCTCGTATGATAACAAGCTATTCAACTCTAGTATCTATCCAC
TTAACGAGAAGTCCTAAATGGGAAAGGCAGACTTCCAATTTTTCAAGTAACTTGCTGTCCTCCTTTATTTTGTC
TTTGTATCATCCAAAAGTCTCTTGTCCCTCCATTTGATCTCTTCCACATTCCTTGTCGCTGCCACTAAATTAAAGA
AGATGCATTTGATTCTGCAATATTATTTTATATTGATGAATCATGTTCATATGTTGTCTGTGGTTTACTTGATCA
GCTTCTTTCCTAGCATGGAAAGATGTAATATAACCGTTTTTGAGTTCTTGCTAAGTTATACTCCCTTCATATCTA
TCAGGTAAAGCTCTCTGGATCCATCAGCAGCCAATACCTGACTGCTCTGCTTATGGCTGCTCCCCTGACTCTAG
GAGATGTGGAGATCGAGATAATTGACAAACTGATATCTGTGCCTTATGTTGAAATGACACTGAAGTTGATGG
AGCGATTTGGTGTCTTTGTGGAGCACAGTAGTGGCTGGGACAGATTCTTGGTAAAAGGAGGTCAGAAGTACA
AGTAAGTCGGCTTTCACACTGTTTTAATGTCTTTAATAATCTGGTTCCACCGATCAATGAAGCTGACAGTTACG
AAAATATCATAGATCTCCTGGGAAAGCATTTGTTGAAGGAGATGCCTCAAGTGCTAGCTATTTTTTGGCGGGG
GCAGCAGTCACAGGTGGAACCGTCACTGTTGAAGGTTGTGGAACAAGCAGTTTACAGGTATTTCTCATGTCAT

TAAGACCTTGGGGGCAATCACATTCTAGACTTTTCCATCTTGTGCTTGTATTTGTGACAGCGCCATAATACGTT
CTATTTCTCTTCTGTTCACTAGCTTAATTTGTGGAAGTACTAAAAAGTAAGAATTGCACAGTGCATGGCCTACA
TCTTAGTGTATAACTGCATAAGGTAATTTTCTAACAAGGGACTTACTTTGAGCTTTATGTTTCCTTTGTGAAGG
GAGATGTTAAGTTCGCTGAGGTCCTCGAGAAGATGGGGGCAGAAGTTACATGGACAGAGAACAGTGTCACA
GTTAAAGGACCTCCGAGGAACTCTTCTGGAATGAAACATTTGCGTGCCATTGACGTGAACATGAACAAAATGC
CAGATGTGGCCATGACTCTTGCCGTAGTTGCACTTTTTGCTGATGGTCCTACTACCATAAGAGATTGGTATGGCT
AACCTTGTTTCCCTGGTATCAAATCCTATTGAAGCACTTCGTTTAGATTAAGATATTATTCTGAAGCGATTGGG
ACCTGGGGCTCTATTCTCCAATATGAAAGCATTTCCCTGCATAATTAGTTTTTTTAAATGCTGTGATAACCTGAC
TAAAATGTGCAAAGAGGTTTTCCTGGTAGTTTAGGGGTCTG

Blue font: 2x TALE binding site (scTALE ABL1 R); yellow highlighted font: G177A; turquoise highlighted font: A268T; red fonts: base changes (without changing a.a coding) for modification of gRNA binding sequences and PAM; orange fonts: red fonts: base changes (without changing a.a coding) for modification of BpiI, BsaI or BsmBI restriction sites for facilitating Golden gate cloning.

- **LbCas12a (wild-type) expression cassette:**

GAATTCCAATCCCACAAAAATCTGAGCTTAACAGCACAGTTGCTCCTCTCAGAGCAGAATCGGGTATTCAACACCC
TCATATCAACTACTACGTTGTGTATAACGGTCCACATGCCGGTATATACGATGACTGGGGTTGTACAAAGGCGGCA
ACAAACGGCGTTCCCGGAGTTGCACACAAGAAATTTGCCACTATTACAGAGGCAAGAGCAGCAGCTGACGCGTAC
ACAACAAGTCAGCAAACAGACAGGTTGAACTTCATCCCCAAAGGAGAAGCTCAACTCAAGCCCAAGAGCTTTGCT
AAGGCCCTAACAAGCCCACCAAAGCAAAAAGCCCACTGGCTCACGCTAGGAACCAAAAGGCCCAGCAGTGATCCA
GCCCCAAAAGAGATCTCCTTTGCCCCGGAGATTACAATGGACGATTTCCTCTATCTTTACGATCTAGGAAGGAAGT
TCGAAGGTGAAGGTGACGACACTATGTTCACCACTGATAATGAGAAGGTTAGCCTCTTCAATTTCAGAAAGAATGC
TGACCCACAGATGGTTAGAGAGGCCTACGCAGCAAGTCTCATCAAGACGATCTACCCGAGTAACAATCTCCAGGA
GATCAAATACCTTCCCAAGAAGGTTAAAGATGCAGTCAAAAGATTCAGGACTAATTGCATCAAGAACACAGAGAA
AGACATATTTCTCAAGATCAGAAGTACTATTCCAGTATGGACGATTCAAGGCTTGCTTCATAAACCAAGGCAAGTA
ATAGAGATTGGAGTCTCTAAAAAGGTAGTTCCTACTGAATCTAAGGCCATGCATGGAGTCTAAGATTCAAATCGAG
GATCTAACAGAACTCGCCGTCAAGACTGGCGAACAGTTCATACAGAGTCTTTTACGACTCAATGACAAGAAGAAA
ATCTTCGTCAACATGGTGGAGCACGACACTCTGGTCTACTCCAAAAATGTCAAAGATACAGTCTCAGAAGATCAAA
GGGCTATTGAGACTTTTCAACAAAGGATAATTTCGGGAAACCTCCTCGGATTCCATTGCCCAGCTATCTGTCACTTC
ATCGAAAGGACAGTAGAAAAGGAAGGTGGCTCCTACAAATGCCATCATTGCGATAAAGGAAAGGCTATCATTCAA
GATCTCTCTGCCGACAGTGGTCCCAAAGATGGACCCCCACCCACGAGGAGCATCGTGGAAAAAGAAGAGGTTCCA
ACCACGTCTACAAAGCAAGTGGATTGATGTGACATCTCCACTGACGTAAGGGATGACGCACAATCCCACTATCCTT
CGCAAGACCCTTCCTCTATATAAGGAAGTTCATTTCATTTGGAGAGGACACGCTCGAGTATAAGGTAAATTTCTGT
GTTCCTTATTCTCTCAAAATCTTCGATTTTGTTTTCGTTCGATCCCAATTTCGTATATGTTCTTTGGTTTAGATTCTGTT
AATCTTAGATCGAAGATGATTTTCTGGGTTTGATCGTTAGATATCATCTTAATTCTCGATTAGGGTTTCATAGATATC
ATCCGATTTGTTCAAATAATTTGAGTTTTGTCGAATAATTACTCTTCGATTTGTGATTTCTATCTAGATCTGGTGTTA
GTTTCTAGTTTGTGCGATCGAATTTGTCGATTAATCTGAGTTTTTCTGATTAACAGGAGCTCATTTTTACAACAATTA
CCAACAACAACAAACAACAAACAACATTACAATTACATTTACAATTATCGATACAATGATGCCCAAGAAGAAGCGC
AAGGTGGGACGCGTCTGCAGGATATCAAGCTTGCGGTACCGCGGGCCCGGGATCGCCACCATGAGCAAGCTGGA
GAAGTTTACAAACTGCTACTCCCTGTCTAAGACCCTGAGGTTCAAGGTAAAGCCTCGATTTTTGGGTTTAGGTGTCT

GCTTATTAGAGTAAAAACACATCCTTTGAAATTGTTTGTGGTCATTTGATTGTGCTCTTGATCCATTGAATTGCTGCA
GGCCATCCCTGTGGGCAAGACCCAGGAGAACATCGACAATAAGCGGCTGCTGGTGGAGGACGAGAAGAGAGCC
GAGGATTATAAGGGCGTGAAGAAGCTGCTGGATCGCTACTATCTGTCTTTTATCAACGACGTGCTGCACAGCATCA
AGCTGAAGAATCTGAACAATTACATCAGCCTGTTCCGGAAGAAAACCAGAACCGAGAAGGAGAATAAGGAGCTG
GAGAACCTGGAGATCAATCTGCGGAAGGAGATCGCCAAGGCCTTCAAGGGCAACGAGGGCTACAAGTCCCTGTT
TAAGAAGGATATCATCGAGACAATCCTGCCAGAGTTCCTGGACGATAAGGACGAGATCGCCCTGGTGAACAGCTT
CAATGGCTTTACCACAGCCTTCACCGGCTTCTTTGATAACAGAGAGAATATGTTTTCCGAGGAGGCCAAGAGCACA
TCCATCGCCTTCAGGTGTATCAACGAGAATCTGACCCGCTACATCTCTAATATGGACATCTTCGAGAAGGTGGACG
CCATCTTTGATAAGCACGAGGTGCAGGAGATCAAGGAGAAGATCCTGAACAGCGACTATGATGTGGAGGATTTCT
TTGAGGGCGAGTTCTTTAACTTTGTGCTGACACAGGAGGGCATCGACGTGTATAACGCCATCATCGGCGGCTTCGT
GACCGAGAGCGGCGAGAAGATCAAGGGCCTGAACGAGTACATCAACCTGTATAATCAGAAAACCAAGCAGAAGC
TGCCTAAGTTTAAGCCACTGTATAAGCAGGTGCTGAGCGATCGGGAGTCTCTGAGCTTCTACGGCGAGGGCTATA
CATCCGATGAGGAGGTGCTGGAGGTGTTTAGAAACACCCTGAACAAGAACAGCGAGATCTTCAGCTCCATCAAGA
AGCTGGAGAAGCTGTTCAAGAATTTTGACGAGTACTCTAGCGCCGGCATCTTTGTGAAGAACGGCCCCGCCATCA
GCACAATCTCCAAGGATATCTTCGGCGAGTGGAACGTGATCCGGGACAAGTGGAATGCCGAGTATGACGATATCC
ACCTGAAGAAGAAGGCCGTGGTGACCGAGAAGTACGAGGACGATCGGAGAAAGTCCTTCAAGAAGATCGGCTCC
TTTTCTCTGGAGCAGCTGCAGGAGTACGCCGACGCCGATCTGTCTGTGGTGGAGAAGCTGAAGGAGATCATCATC
CAGAAGGTGGATGAGATCTACAAGGTGTATGGCTCCTCTGAGAAGCTGTTCGACGCCGATTTTGTGCTGGAGAAG
AGCCTGAAGAAGAACGACGCCGTGGTGGCCATCATGAAGGACCTGCTGGATTCTGTGAAGAGCTTCGAGAATTAC
ATCAAGGCCTTCTTTGGCGAGGGCAAGGAGACAAACAGGGACGAGTCCTTCTATGGCGATTTTGTGCTGGCCTAC
GACATCCTGCTGAAGGTGGACCACATCTACGATGCCATCCGCAATTATGTGACCCAGAAGCCCTACTCTAAGGATA
AGTTCAAGCTGTATTTTCAGAACCCTCAGTTCATGGGCGGCTGGGACAAGGATAAGGAGACAGACTATCGGGCCA
CCATCCTGAGATACGGCTCCAAGTACTATCTGGCCATCATGGATAAGAAGTACGCCAAGTGCCTGCAGAAGATCG
ACAAGGACGATGTGAACGGCAATTACGAGAAGATCAACTATAAGCTGCTGCCCGGCCCTAATAAGATGCTGCCAA
AGGTGTTCTTTTCTAAGAAGTGGATGGCCTACTATAACCCCAGCGAGGACATCCAGAAGATCTACAAGAATGGCA
CATTCAAGAAGGGCGATATGTTTAACCTGAATGACTGTCACAAGCTGATCGACTTCTTTAAGGATAGCATCTCCCG
GTATCCAAAGTGGTCCAATGCCTACGATTTCAACTTTTCTGAGACAGAGAAGTATAAGGACATCGCCGGCTTTTAC
AGAGAGGTGGAGGAGCAGGGCTATAAGGTGAGCTTCGAGTCTGCCAGCAAGAAGGAGGTGGATAAGCTGGTGG
AGGAGGGCAAGCTGTATATGTTCCAGATCTATAACAAGGACTTTTCCGATAAGTCTCACGGCACACCCAATCTGCA
CACCATGTACTTCAAGCTGCTGTTTGACGAGAACAATCACGGACAGATCAGGCTGAGCGGAGGAGCAGAGCTGTT
CATGAGGCGCGCCTCCCTGAAGAAGGAGGAGCTGGTGGTGCACCCAGCCAACTCCCCTATCGCCAACAAGAATCC
AGATAATCCCAAGAAAACCACAACCCTGTCCTACGACGTGTATAAGGATAAGAGGTTTTCTGAGGACCAGTACGA
GCTGCACATCCCAATCGCCATCAATAAGTGCCCCAAGAACATCTTCAAGATCAATACAGAGGTGCGCGTGCTGCTG
AAGCACGACGATAACCCCTATGTGATCGGCATCGATAGGGGCGAGCGCAATCTGCTGTATATCGTGGTGGTGGAC
GGCAAGGGCAACATCGTGGAGCAGTATTCCCTGAACGAGATCATCAACAACTTCAACGGCATCAGGATCAAGACA
GATTACCACTCTCTGCTGGACAAGAAGGAGAAGGAGAGGTTCGAGGCCCGCCAGAACTGGACCTCCATCGAGAA
TATCAAGGAGCTGAAGGCCGGCTATATCTCTCAGGTGGTGCACAAGATCTGCGAGCTGGTGGAGAAGTACGATG
CCGTGATCGCCCTGGAGGACCTGAACTCTGGCTTTAAGAATAGCCGCGTGAAGGTGGAGAAGCAGGTGTATCAG
AAGTTCGAGAAGATGCTGATCGATAAGCTGAACTACATGGTGGACAAGAAGTCTAATCCTTGTGCAACAGGCGGC
GCCCTGAAGGGCTATCAGATCACCAATAAGTTCGAGAGCTTTAAGTCCATGTCTACCCAGAACGGCTTCATCTTTTA
CATCCCTGCCTGGCTGACATCCAAGATCGATCCATCTACCGGCTTTGTGAACCTGCTGAAAACCAAGTATACCAGC
ATCGCCGATTCCAAGAAGTTCATCAGCTCCTTTGACAGGATCATGTACGTGCCCGAGGAGGATCTGTTCGAGTTTG

CCCTGGACTATAAGAACTTCTCTCGCACAGACGCCGATTACATCAAGAAGTGGAAGCTGTACTCCTACGGCAACCG
GATCAGAATCTTCCGGAATCCTAAGAAGAACAACGTGTTCGACTGGGAGGAGGTGTGCCTGACCAGCGCCTATAA
GGAGCTGTTCAACAAGTACGGCATCAATTATCAGCAGGGCGATATCAGAGCCCTGCTGTGCGAGCAGTCCGACAA
GGCCTTCTACTCTAGCTTTATGGCCCTGATGAGCCTGATGCTGCAGATGCGGAACAGCATCACAGGCCGCACCGAC
GTGGATTTTCTGATCAGCCCTGTGAAGAACTCCGACGGCATCTTCTACGATAGCCGGAACTATGAGGCCCAGGAG
AATGCCATCCTGCCAAAGAACGCCGACGCCAATGGCGCCTATAACATCGCCAGAAAGGTGCTGTGGGCCATCGGC
CAGTTCAAGAAGGCCGAGGACGAGAAGCTGGATAAGGTGAAGATCGCCATCTCTAACAAGGAGTGGCTGGAGTA
CGCCCAGACCAGCGTGAAGCACGCCTATCCCTATGACGTGCCCGATTATGCCAGCCTGGGCAGCGGCTCCCCCAA
GAAAAAACGCAAGGTGGAAGATCCTAAGAAAAAGCGGAAAGTGGACGGCATTGGTAGTGGGAGCTAAGCTTCTC
TAGCTAGAGTCGATCGACAAGCTCGAGTTTCTCCATAATAATGTGTGAGTAGTTCCCAGATAAGGGAATTAGGGTT
CCTATAGGGTTTCGCTCATGTGTTGAGCATATAAGAAACCCTTAGTATGTATTTGTATTTGTAAAATACTTCTATCAA
TAAAATTTCTAATTCCTAAAACCAAAATCCAGTACTAAAATCCAGATC

Purple font: SV40 NLS; blue font: LbCas12a; green font: HA tag; orange font: CaMV 35S promoter; tan font: Trp1 intron; dark blue: AtUBQ10 intron 1; black font: linkers.

❖ **ttLbCas12a (temperature-tolerant) expression cassette:**

GAATTCCAATCCCACAAAAATCTGAGCTTAACAGCACAGTTGCTCCTCTCAGAGCAGAATCGGGTATTCAACACCC
TCATATCAACTACTACGTTGTGTATAACGGTCCACATGCCGGTATATACGATGACTGGGGTTGTACAAAGGCGGCA
ACAAACGGCGTTCCCGGAGTTGCACACAAGAAATTTGCCACTATTACAGAGGCAAGAGCAGCAGCTGACGCGTAC
ACAACAAGTCAGCAAACAGACAGGTTGAACTTCATCCCCAAAGGAGAAGCTCAACTCAAGCCCAAGAGCTTTGCT
AAGGCCCTAACAAGCCCACCAAAGCAAAAAGCCCACTGGCTCACGCTAGGAACCAAAAGGCCCAGCAGTGATCCA
GCCCCAAAAGAGATCTCCTTTGCCCCGGAGATTACAATGGACGATTTCCTCTATCTTTACGATCTAGGAAGGAAGT
TCGAAGGTGAAGGTGACGACACTATGTTCACCACTGATAATGAGAAGGTTAGCCTCTTCAATTTCAGAAAGAATGC
TGACCCACAGATGGTTAGAGAGGCCTACGCAGCAAGTCTCATCAAGACGATCTACCCGAGTAACAATCTCCAGGA
GATCAAATACCTTCCCAAGAAGGTTAAAGATGCAGTCAAAAGATTCAGGACTAATTGCATCAAGAACACAGAGAA
AGACATATTTCTCAAGATCAGAAGTACTATTCCAGTATGGACGATTCAAGGCTTGCTTCATAAACCAAGGCAAGTA
ATAGAGATTGGAGTCTCTAAAAAGGTAGTTCCTACTGAATCTAAGGCCATGCATGGAGTCTAAGATTCAAATCGAG
GATCTAACAGAACTCGCCGTCAAGACTGGCGAACAGTTCATACAGAGTCTTTTACGACTCAATGACAAGAAGAAA
ATCTTCGTCAACATGGTGGAGCACGACACTCTGGTCTACTCCAAAAATGTCAAAGATACAGTCTCAGAAGATCAAA
GGGCTATTGAGACTTTTCAACAAAGGATAATTTCGGGAAACCTCCTCGGATTCCATTGCCCAGCTATCTGTCACTTC
ATCGAAAGGACAGTAGAAAAGGAAGGTGGCTCCTACAAATGCCATCATTGCGATAAAGGAAAGGCTATCATTCAA
GATCTCTCTGCCGACAGTGGTCCCAAAGATGGACCCCCACCCACGAGGAGCATCGTGGAAAAAGAAGAGGTTCCA
ACCACGTCTACAAAGCAAGTGGATTGATGTGACATCTCCACTGACGTAAGGGATGACGCACAATCCCACTATCCTT
CGCAAGACCCTTCCTCTATATAAGGAAGTTCATTTCATTTGGAGAGGACACGCTCGAGTATAAGGTAAATTTCTGT
GTTCCTTATTCTCTCAAAATCTTCGATTTTGTTTTCGTTCGATCCCAATTTCGTATATGTTCTTTGGTTTAGATTCTGTT
AATCTTAGATCGAAGATGATTTTCTGGGTTTGATCGTTAGATATCATCTTAATTCTCGATTAGGGTTTCATAGATATC
ATCCGATTTGTTCAAATAATTTGAGTTTTGTCGAATAATTACTCTTCGATTTGTGATTTCTATCTAGATCTGGTGTTA
GTTTCTAGTTTGTGCGATCGAATTTGTCGATTAATCTGAGTTTTTCTGATTAACAGGAGCTCATTTTTACAACAATTA
CCAACAACAACAAACAACAAACAACATTACAATTACATTTACAATTATCGATACAATGATGCCCAAGAAGAAGCGC
AAGGTGGGACGCGTCTGCAGGATATCAAGCTTGCGGTACCGCGGGCCCGGGATCGCCACCATGAGCAAGCTGGA
GAAGTTTACAAACTGCTACTCCCTGTCTAAGACCCTGAGGTTCAAGGTAAAGCCTCGATTTTTGGGTTTAGGTGTCT

GCTTATTAGAGTAAAAACACATCCTTTGAAATTGTTTGTGGTCATTTGATTGTGCTCTTGATCCATTGAATTGCTGCA
GGCCATCCCTGTGGGCAAGACCCAGGAGAACATCGACAATAAGCGGCTGCTGGTGGAGGACGAGAAGAGAGCC
GAGGATTATAAGGGCGTGAAGAAGCTGCTGGATCGCTACTATCTGTCTTTTATCAACGACGTGCTGCACAGCATCA
AGCTGAAGAATCTGAACAATTACATCAGCCTGTTCCGGAAGAAAACCAGAACCGAGAAGGAGAATAAGGAGCTG
GAGAACCTGGAGATCAATCTGCGGAAGGAGATCGCCAAGGCCTTCAAGGGCAACGAGGGCTACAAGTCCCTGTT
TAAGAAGGATATCATCGAGACAATCCTGCCAGAGTTCCTGGACGATAAGGACGAGATCGCCCTGGTGAACAGCTT
CAATGGCTTTACCACAGCCTTCACCGGCTTCTTTAGAAACAGAGAGAATATGTTTTCCGAGGAGGCCAAGAGCACA
TCCATCGCCTTCAGGTGTATCAACGAGAATCTGACCCGCTACATCTCTAATATGGACATCTTCGAGAAGGTGGACG
CCATCTTTGATAAGCACGAGGTGCAGGAGATCAAGGAGAAGATCCTGAACAGCGACTATGATGTGGAGGATTTCT
TTGAGGGCGAGTTCTTTAACTTTGTGCTGACACAGGAGGGCATCGACGTGTATAACGCCATCATCGGCGGCTTCGT
GACCGAGAGCGGCGAGAAGATCAAGGGCCTGAACGAGTACATCAACCTGTATAATCAGAAAACCAAGCAGAAGC
TGCCTAAGTTTAAGCCACTGTATAAGCAGGTGCTGAGCGATCGGGAGTCTCTGAGCTTCTACGGCGAGGGCTATA
CATCCGATGAGGAGGTGCTGGAGGTGTTTAGAAACACCCTGAACAAGAACAGCGAGATCTTCAGCTCCATCAAGA
AGCTGGAGAAGCTGTTCAAGAATTTTGACGAGTACTCTAGCGCCGGCATCTTTGTGAAGAACGGCCCCGCCATCA
GCACAATCTCCAAGGATATCTTCGGCGAGTGGAACGTGATCCGGGACAAGTGGAATGCCGAGTATGACGATATCC
ACCTGAAGAAGAAGGCCGTGGTGACCGAGAAGTACGAGGACGATCGGAGAAAGTCCTTCAAGAAGATCGGCTCC
TTTTCTCTGGAGCAGCTGCAGGAGTACGCCGACGCCGATCTGTCTGTGGTGGAGAAGCTGAAGGAGATCATCATC
CAGAAGGTGGATGAGATCTACAAGGTGTATGGCTCCTCTGAGAAGCTGTTCGACGCCGATTTTGTGCTGGAGAAG
AGCCTGAAGAAGAACGACGCCGTGGTGGCCATCATGAAGGACCTGCTGGATTCTGTGAAGAGCTTCGAGAATTAC
ATCAAGGCCTTCTTTGGCGAGGGCAAGGAGACAAACAGGGACGAGTCCTTCTATGGCGATTTTGTGCTGGCCTAC
GACATCCTGCTGAAGGTGGACCACATCTACGATGCCATCCGCAATTATGTGACCCAGAAGCCCTACTCTAAGGATA
AGTTCAAGCTGTATTTTCAGAACCCTCAGTTCATGGGCGGCTGGGACAAGGATAAGGAGACAGACTATCGGGCCA
CCATCCTGAGATACGGCTCCAAGTACTATCTGGCCATCATGGATAAGAAGTACGCCAAGTGCCTGCAGAAGATCG
ACAAGGACGATGTGAACGGCAATTACGAGAAGATCAACTATAAGCTGCTGCCCGGCCCTAATAAGATGCTGCCAA
AGGTGTTCTTTTCTAAGAAGTGGATGGCCTACTATAACCCCAGCGAGGACATCCAGAAGATCTACAAGAATGGCA
CATTCAAGAAGGGCGATATGTTTAACCTGAATGACTGTCACAAGCTGATCGACTTCTTTAAGGATAGCATCTCCCG
GTATCCAAAGTGGTCCAATGCCTACGATTTCAACTTTTCTGAGACAGAGAAGTATAAGGACATCGCCGGCTTTTAC
AGAGAGGTGGAGGAGCAGGGCTATAAGGTGAGCTTCGAGTCTGCCAGCAAGAAGGAGGTGGATAAGCTGGTGG
AGGAGGGCAAGCTGTATATGTTCCAGATCTATAACAAGGACTTTTCCGATAAGTCTCACGGCACACCCAATCTGCA
CACCATGTACTTCAAGCTGCTGTTTGACGAGAACAATCACGGACAGATCAGGCTGAGCGGAGGAGCAGAGCTGTT
CATGAGGCGCGCCTCCCTGAAGAAGGAGGAGCTGGTGGTGCACCCAGCCAACTCCCCTATCGCCAACAAGAATCC
AGATAATCCCAAGAAAACCACAACCCTGTCCTACGACGTGTATAAGGATAAGAGGTTTTCTGAGGACCAGTACGA
GCTGCACATCCCAATCGCCATCAATAAGTGCCCCAAGAACATCTTCAAGATCAATACAGAGGTGCGCGTGCTGCTG
AAGCACGACGATAACCCCTATGTGATCGGCATCGATAGGGGCGAGCGCAATCTGCTGTATATCGTGGTGGTGGAC
GGCAAGGGCAACATCGTGGAGCAGTATTCCCTGAACGAGATCATCAACAACTTCAACGGCATCAGGATCAAGACA
GATTACCACTCTCTGCTGGACAAGAAGGAGAAGGAGAGGTTCGAGGCCCGCCAGAACTGGACCTCCATCGAGAA
TATCAAGGAGCTGAAGGCCGGCTATATCTCTCAGGTGGTGCACAAGATCTGCGAGCTGGTGGAGAAGTACGATG
CCGTGATCGCCCTGGAGGACCTGAACTCTGGCTTTAAGAATAGCCGCGTGAAGGTGGAGAAGCAGGTGTATCAG
AAGTTCGAGAAGATGCTGATCGATAAGCTGAACTACATGGTGGACAAGAAGTCTAATCCTTGTGCAACAGGCGGC
GCCCTGAAGGGCTATCAGATCACCAATAAGTTCGAGAGCTTTAAGTCCATGTCTACCCAGAACGGCTTCATCTTTTA
CATCCCTGCCTGGCTGACATCCAAGATCGATCCATCTACCGGCTTTGTGAACCTGCTGAAAACCAAGTATACCAGC
ATCGCCGATTCCAAGAAGTTCATCAGCTCCTTTGACAGGATCATGTACGTGCCCGAGGAGGATCTGTTCGAGTTTG

CCCTGGACTATAAGAACTTCTCTCGCACAGACGCCGATTACATCAAGAAGTGGAAGCTGTACTCCTACGGCAACCG
GATCAGAATCTTCCGGAATCCTAAGAAGAACAACGTGTTCGACTGGGAGGAGGTGTGCCTGACCAGCGCCTATAA
GGAGCTGTTCAACAAGTACGGCATCAATTATCAGCAGGGCGATATCAGAGCCCTGCTGTGCGAGCAGTCCGACAA
GGCCTTCTACTCTAGCTTTATGGCCCTGATGAGCCTGATGCTGCAGATGCGGAACAGCATCACAGGCCGCACCGAC
GTGGATTTTCTGATCAGCCCTGTGAAGAACTCCGACGGCATCTTCTACGATAGCCGGAACTATGAGGCCCAGGAG
AATGCCATCCTGCCAAAGAACGCCGACGCCAATGGCGCCTATAACATCGCCAGAAAGGTGCTGTGGGCCATCGGC
CAGTTCAAGAAGGCCGAGGACGAGAAGCTGGATAAGGTGAAGATCGCCATCTCTAACAAGGAGTGGCTGGAGTA
CGCCCAGACCAGCGTGAAGCACGCCTATCCCTATGACGTGCCCGATTATGCCAGCCTGGGCAGCGGCTCCCCCAA
GAAAAAACGCAAGGTGGAAGATCCTAAGAAAAAGCGGAAAGTGGACGGCATTGGTAGTGGGAGCTAAGCTTCTC
TAGCTAGAGTCGATCGACAAGCTCGAGTTTCTCCATAATAATGTGTGAGTAGTTCCCAGATAAGGGAATTAGGGTT
CCTATAGGGTTTCGCTCATGTGTTGAGCATATAAGAAACCCTTAGTATGTATTTGTATTTGTAAAATACTTCTATCAA
TAAAATTTCTAATTCCTAAAACCAAAATCCAGTACTAAAATCCAGATC

Purple font: SV40 NLS; blue font: LbCas12a; green font: HA tag; orange font: CaMV 35S promoter; tan font: Trp1 intron; dark blue: AtUBQ10 intron 1; yellow highlighted font: D156R modification of the temperature tolerant LbCas12a; black font: linkers.

- **SpCas9 expression cassette**: the p35S-SpCas9-ter was cloned from plasmid pICH47742::2x35S-5’UTR-hCas9(STOP)-NOST (Addgene Plasmid #49771).

- **Plant selection marker**: pNOS-NptII-tOCS cloned from pICSL11024 (pICH47732::NOSp-NPTII-OCST) (Addgene Plasmid #51144).
